# Study on the Dynamic Characteristics of Rub-Impact and Bearing Defect Coupled Faults in a Single-Disk Double-Bearing Rotor System

**DOI:** 10.3390/ma19132798

**Published:** 2026-07-01

**Authors:** Junming Liu, Hongyuan Zhang, Hongyun Sun, He Wang, Zhuan Chang

**Affiliations:** School of Automotive and Transportation, Shenyang Ligong University, Shenyang 110159, China; 2402620186@stu.sylu.edu.cn (J.L.); shy5006@sylu.edu.cn (H.S.); 2302610196@stu.sylu.edu.cn (H.W.); zchang@sylu.edu.cn (Z.C.)

**Keywords:** rotor dynamics, rub-impact fault, rub-impact stiffness, eccentricity, bearing ring defect width

## Abstract

Rub-impact is a critical failure mode in high-speed rotor systems that heavily complicates fault diagnosis. While traditionally studied in aero-engines due to its severe risks of blade damage and thermal-induced rotor instability, rub-impact has increasingly emerged as a crucial concern in modern electric vehicle (EV) traction motors characterized by high speeds, slender shafts, and ultra-narrow rotor–stator air gaps. Since rub-impact rarely occurs in isolation, this study establishes a dynamic model of an EV motor rotor system experiencing compound rub-impact and bearing faults based on Jeffcott rotor theory and the lumped-mass method. The influences of key fault parameters on system dynamics are comprehensively investigated through analyses of time histories, phase trajectories, Poincaré sections, frequency spectra, and envelope spectra. The results show that increasing the rub-impact stiffness (from 1.0 × 10^10^ N/m to 3.0 × 10^10^ N/m) significantly enhances the non-linear impulsive behavior of the system while reducing the rotor unbalance vibration amplitude by 20.0%. Under compound fault conditions with a local bearing defect width of 3 mm, the disk response is mainly governed by global rub-impact behavior, whereas the bearing-end response is more sensitive to local bearing defects. Under compound fault conditions, although widening the localized bearing defect (from 1 mm to 3 mm) significantly exacerbates the local fault severity at the bearing end, the disk’s phase trajectories, Poincaré maps, and spectra remain virtually uninfluenced. This is attributed to the fact that the relative signature intensity of the bearing fault characteristic frequency $f_{i}$ attenuates by more than 99% during structural transmission, causing the global non-linear dynamics of the rotor disk to be exclusively governed by global rub-impact behavior and completely insensitive to the localized defect propagation. These quantitative findings provide a precise theoretical basis for the diagnosis and identification of compound faults in rotor systems.

## 1. Introduction

Rolling bearings are critical yet highly vulnerable mechanical components in modern rotating machinery. Statistical evidence indicates that the main causes of rolling bearing failure typically stem from rolling contact fatigue (RCF), inadequate or contaminated lubrication, misalignment, and excessive operational loads. Among various configurations, the selected roller bearings in high-speed and heavy-duty applications are frequently identified as the elements that fail most often, primarily due to localized stress concentration, subsurface-initiated spalling, and severe rub-impact induced by non-linear compound faults. To resolve these failure issues and enhance system reliability, several engineering methods are widely adopted, including the optimization of bearing internal clearance, implementation of advanced early-stage fault diagnostics, and utilization of high-strength actinide-alloyed or vacuum-remelted steels. Crucially, understanding these multi-physical failure mechanisms and data-driven quantification under stochastic uncertainties remains paramount for the health monitoring of complex rotor–bearing systems [1].

To mathematically bound the high-speed operational responses, various historical and state-of-the-art modeling frameworks have been established sequentially. Specifically, Li et al. [2] proposed a dedicated fault diagnosis index to capture the kinematic characteristics triggered by fixed-point rub-impact behaviors. To account for fluid-boundary constraints, Nan et al. [3] rigorously formulated the non-linear oil-film forces to evaluate the rubbing dynamics of dual-disk systems supported by hydrodynamic journal bearings. Furthermore, addressing complex multi-body structural configurations, Zhu et al. [4] utilized localized matrices to investigate the rub-impact behavior of bolted joint rotor–bearing systems considering interface hysteresis, while Wang et al. [5] successfully implemented the finite element method (FEM) and lumped-discretization schemes to map the general fault modeling and vibration characteristics of high-speed turbocharger rotors.

To solve the resulting high-dimensional and non-continuous equations of motion, various numerical integration schemes and solution algorithms have been widely implemented. Specifically, Zhang et al. [6] successfully utilized the adaptive Runge–Kutta integration scheme to solve the non-linear transient governing equations for early-stage rub-impact energy tracking. To provide a high-level methodological taxonomy, Prabith and Krishna [7] compiled a comprehensive review that systematically categorized the state-of-the-art numerical modeling and discretization schemes used in rotor–stator rubbing analysis. Furthermore, Lu et al. [8] implemented a time-stepping ODE numerical solver combined with non-linear output frequency response functions (NOFRFs) to achieve quantitative fault feature extraction, while Wang et al. [9] deployed the Fourth-order Runge–Kutta method to perform transient state analysis of a complex rub-impact rotor system during maneuvering flights.

In recent years, extensive research has been conducted on the dynamic modeling, non-linear response analysis, and fault diagnosis of rub-impact faults, with significant progress achieved in finite element modeling, non-linear oil-film force analysis, vibration energy trajectory methods, and NOFRFs-based feature extraction. Meanwhile, studies on numerical models of rub-impact faults and non-linear dynamic behaviors under complex operating conditions have been further developed, providing an important theoretical basis for mechanism analysis and diagnosis of rotor rub-impact faults.

As an important supporting component of rotor systems, rolling bearings significantly affect system stability when inner- and outer-race faults occur. Current research on bearing failures mainly focuses on fatigue fracture, wear degradation, thermal failure, and lubrication conditions. Through metallurgical analysis, vibration testing, and numerical simulation, the failure mechanisms and life degradation laws of bearings have been systematically investigated [10,11,12,13]. In addition, studies on contact stress, thermal error prediction, and the influence of temperature on fault characteristic frequency drift further reveal the dynamic evolution characteristics of bearings under complex operating conditions [14,15,16,17,18].

In recent years, research on compound fault dynamics has become a major focus. Existing studies have investigated coupled faults involving rub-impact with cracks, looseness, misalignment, and oil-film instability, revealing complex non-linear dynamic behaviors such as bifurcation, chaos, low-frequency amplification, and spectral coupling under multi-fault interactions [19,20,21,22]. Moreover, research on flexible rotor–bearing coupled systems, multi-fault energy interaction mechanisms, and the coupling effects of fluid-film forces and rub-impact has provided important insights for compound fault identification and system stability analysis [23,24,25,26,27].

At present, rotor dynamics are commonly studied using Jeffcott rotor theory, Euler–Bernoulli beam models, multi-disk rotor models, and finite element models [28,29,30,31]. This study is based on the Jeffcott rotor theory, in which a lumped-mass dynamic model is developed and the corresponding equations of motion are established. A systematic investigation is conducted on the compound operating conditions involving rub-impact faults and bearing inner- and outer-race defects. Rather than constructing an anatomically perfect multi-physical replica, this work utilizes a geometrically simplified approach to provide a preliminary quantitative reference and practical parametric baseline regarding the transmission attenuation mechanisms of multi-fault coupled dynamics. Crucially, the mathematical tracking of such non-linear topologies is heavily built upon the time-stepping convergence algorithms and non-linear bifurcation analysis paradigms previously established by our research group [32,33,34], which serve as the direct methodological baseline and solver stability support for the transient fault-decoupling formulation in the present work.

The specific significance of this paper and its distinct novelty that sets it apart from previous studies on similar topics lie in the rigorous quantification of the non-linear coupling mechanisms within a multi-fault rotor system. Compared with the existing literature that often analyzes rub-impact or bearing defects in isolation, this work establishes a comprehensive compound fault model to reveal how global rub-impact behavior modulates and masks localized bearing signatures. This paper is highly worthy of publication in this journal because it bridges the gap between theoretical non-linear rotor dynamics and practical engineering diagnostics through the following three distinct contributions: (i) it presents a coupled dynamic model based on the lumped-mass method that accurately captures the interactive feedback forces between localized bearing propagation and global boundary constraints; (ii) it identifies a critical physical phenomenon where the relative signature intensity of the bearing fault characteristic frequency attenuates by more than 99% during structural transmission, providing a quantitative explanation for why local defects are easily masked; and (iii) it offers precise numerical thresholds (e.g., rub-impact stiffness ranges and defect widths) that directly serve as a theoretical benchmark for the early-stage health monitoring and multi-fault decoupling of aero-engines and electric vehicle traction motors. Section 1 provides a systematic review of recent domestic and international advances in rotor system dynamics. Section 2 establishes the dynamic models of the rotor system under both healthy and faulty conditions. Section 3 systematically analyzes the resulting dynamic characteristics under single and compound fault configurations. Finally, Section 4 summarizes the overall research work and highlights the main conclusions of this study.

## 2. Dynamic Modeling of the Rolling Bearing–Rotor System

### 2.1. Dynamic Modeling of the Healthy Rolling Bearing–Rotor System

**Figure 1:**
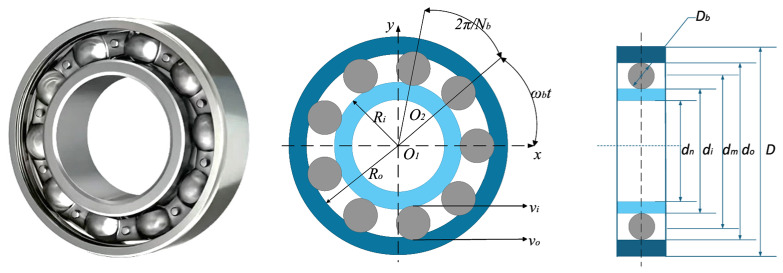
Schematic diagram of rolling bearing.

In this study, a specific type of rolling bearing is selected as the research object. The following fundamental assumptions are introduced in establishing its dynamic model: all structural components of the bearing are considered rigid bodies, and the effects of material contact deformation and oil-film lubrication are neglected. It is assumed that the rolling elements are uniformly distributed between the inner and outer raceways, and that all rolling elements undergo pure rolling motion, with their surface linear velocities being fully consistent with the linear velocities at the contact points of the inner and outer rings. The simplified structural model of the rolling bearing constructed based on the above theoretical assumptions is shown in Figure 1.

It is further assumed that the outer ring of the bearing is rigidly connected to the bearing housing, while the inner ring is rigidly connected to the shaft. The rolling elements are maintained at equal spacing between the inner and outer raceways by the cage. To enhance readability, a detailed nomenclature defining all mathematical symbols, subscripts, and units used in this dynamic framework is provided at the end of the manuscript. Under these conditions, the linear velocities $v_{i}$ and $v_{o}$ at the contact points between the rolling elements and the inner and outer rings can be expressed by Equation (1), respectively.

(1) $$v_{i}=\omega_{i}\cdot R_{i}\;\;\;\;v_{o}=\omega_{o}\cdot R_{o}$$

Under the condition that the cage and the rolling elements maintain the same orbital angular velocity, the orbital linear velocity $v_{b}$ at the center of the cage can be expressed by Equation (2):

(2) $$v_{c}=\frac{\left(v_{o}+v_{i}\right)}{2}$$

Since the bearing outer ring is fixed to the bearing housing and the inner ring is fixed to the rotating shaft, it follows that $\omega_{o}=0$ and $\omega_{i}=\omega$. Therefore, the angular velocity of the cage, $\omega_{c}$, can be expressed as:

(3) $$\omega_{c}=2\cdot \frac{v_{c}}{\left(R_{o}+R_{i}\right)}=\omega \cdot \frac{R_{i}}{\left(R_{o}+R_{i}\right)}$$

The rotational angular position of the i-th rolling element within time t can be expressed by Equation (4):

(4) $$\varphi_{i}=\omega_{c}t+\frac{2\pi (i-1)}{N_{b}}\;\;\;\;i=1,2,\cdots ,N_{b}$$

During the operation of a rolling bearing, the radial load acting on the rolling elements varies periodically with their angular positions, thereby inducing vibration responses with characteristic frequency components. This phenomenon is referred to as rolling element passage vibration, also known as VC vibration, and can be expressed as:

(5) $$f_{vc}=\omega_{c}\times N_{b}$$

The angular position of the i-th rolling element, denoted by $\theta_{i}$, can be expressed as:

(6) $$\theta_{i}=\omega_{c}t+\frac{2\pi }{N_{b}}(i-1)$$

The elastic deformation at the contact point can be expressed as:

(7) $$r_{\theta_{i}}=\frac{2\Gamma }{\pi }{\left(\frac{3Q^{2}\Sigma \rho }{8}{\left(\frac{1-\nu^{2}}{E}\right)}^{2}\left(\frac{\pi k^{2}}{2\Sigma }\right)\right)}^{\frac{1}{3}}$$

The contact stiffness of the rolling bearing can be obtained from Equation (8).

(8) $$k_{b}=\frac{2E}{3(1-\nu^{2})}\sqrt{\frac{2}{{\left(\frac{2\Gamma k}{\Sigma \pi }\right)}^{3}\Sigma \rho }}$$

According to the schematic, the elastic deformation of the contact between the i-th rolling element and the inner and outer raceways at any time can be expressed as follows:

(9) $$r_{\theta_{i}}=x\cos\theta_{i}+y\sin\theta_{i}-\gamma$$

By summing the contact forces acting on each rolling element, the total Hertzian contact forces of the left and right bearings in the x- and y-directions can be obtained:

(10) $$\left\{\begin{array}{l} f_{xb1}=-\sum_{i=1}^{N_{b}}k_{bb1}{\left[(x_{b1}\cos\theta_{i}+y_{b1}\sin\theta_{i})-\gamma \right]}_{+}^{1.5}\cos\theta_{i}\\ f_{yb1}=-\sum_{i=1}^{N_{b}}k_{bb1}{\left[(x_{b1}\cos\theta_{i}+y_{b1}\sin\theta_{i})-\gamma \right]}_{+}^{1.5}\sin\theta_{i}\\ f_{xb2}=-\sum_{i=1}^{N_{b}}k_{bb2}{\left[(x_{b2}\cos\theta_{i}+y_{b2}\sin\theta_{i})-\gamma \right]}_{+}^{1.5}\cos\theta_{i}\\ f_{yb2}=-\sum_{i=1}^{N_{b}}k_{bb2}{\left[(x_{b2}\cos\theta_{i}+y_{b2}\sin\theta_{i})-\gamma \right]}_{+}^{1.5}\sin\theta_{i} \end{array}\right.$$

In the equation, $N_{b}$ denotes the number of rolling elements, and the superscript “+” represents the positive-part operator. When the expression inside the parentheses yields a positive value, the rolling element located at angular position $\theta_{i}$ is within the load-carrying zone and contributes an incremental contact force to the total load; when the result is zero or negative, the rolling element lies in the non-load-carrying zone and its Hertzian contact force is taken as zero.

**Figure 2:**
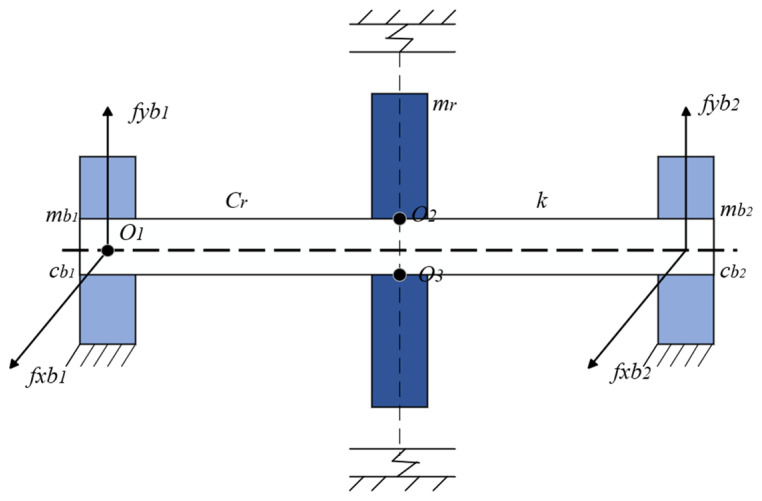
Rolling bearing–rotor system model.

In this study, a simplified dynamic model of the rolling bearing–rotor system is developed. The system is reduced to three lumped-mass units, with only translational degrees of freedom in the x- and y-directions considered for analysis. All bearing components are assumed to be rigid bodies. Based on these assumptions, the schematic diagram of the six-degree-of-freedom system is shown in Figure 2.

The non-linear equations of motion of the system are given as follows:

(11) $$\left\{\begin{array}{l} m_{b1}{\ddot{x}}_{b1}+c_{b1}{\dot{x}}_{b1}+k(x_{b1}-x_{r})=f_{bx1}\\ m_{b1}{\ddot{y}}_{b1}+c_{b1}{\dot{y}}_{b1}+k(y_{b1}-y_{r})=f_{by1}-m_{b1}g\\ m_{r}{\ddot{x}}_{r}+c_{r}{\dot{x}}_{r}+k(x_{r}-x_{b1})+k(x_{r}-x_{b2})=m_{r}e\omega^{2}\cos(\omega t)\\ m_{r}{\ddot{y}}_{r}+c_{r}{\dot{y}}_{r}+k(y_{r}-y_{b1})+k(y_{r}-y_{b2})=m_{r}e\omega^{2}\sin(\omega t)-m_{r}g\\ m_{b2}{\ddot{x}}_{b2}+c_{b2}{\dot{x}}_{b2}+k(x_{b2}-x_{r})=f_{bx2}\\ m_{b2}{\ddot{y}}_{b2}+c_{b2}{\dot{y}}_{b2}+k(y_{b2}-y_{r})=f_{by2}-m_{b2}g \end{array}\right.$$

To simplify the calculations and unify the parameters, the governing differential equations of the system are nondimensionalized by introducing the following dimensionless variables:

(12) $$\left\{\begin{array}{l} \tau =\omega t,C_{r}=\frac{c_{r}}{m_{r}\omega },C_{b1}=\frac{c_{b1}}{m_{b1}\omega },C_{b2}=\frac{c_{b2}}{m_{b2}\omega }\\ K=\frac{k}{m_{b1}\omega },K_{bb1}=\frac{k_{b}\sqrt{\gamma }}{m_{b1}\omega },K_{bb2}=\frac{k_{b}\sqrt{\gamma }}{m_{b2}\omega },G=\frac{g}{\gamma \omega^{2}}\\ E=\frac{e}{\gamma },X_{r}=\frac{x_{r}}{\gamma },Y_{r}=\frac{y_{r}}{\gamma },X_{b1}=\frac{x_{b1}}{\gamma },Y_{b1}=\frac{y_{b1}}{\gamma }\\ X_{b2}=\frac{x_{b2}}{\gamma },Y_{b2}=\frac{y_{b2}}{\gamma },{\dot{X}}_{r}=\frac{{\dot{x}}_{r}}{\gamma \omega },{\dot{Y}}_{r}=\frac{{\dot{y}}_{r}}{\gamma \omega },{\dot{X}}_{b1}=\frac{{\dot{x}}_{b1}}{\gamma \omega }\\ {\dot{Y}}_{b1}=\frac{{\dot{y}}_{b1}}{\gamma \omega },{\dot{X}}_{b2}=\frac{{\dot{x}}_{b2}}{\gamma \omega },{\dot{Y}}_{b2}=\frac{{\dot{y}}_{b2}}{\gamma \omega },{\ddot{X}}_{r}=\frac{{\ddot{x}}_{r}}{\gamma \omega^{2}},{\ddot{Y}}_{r}=\frac{{\ddot{y}}_{r}}{\gamma \omega^{2}}\\ {\ddot{X}}_{b1}=\frac{{\ddot{x}}_{b1}}{\gamma \omega^{2}},{\ddot{Y}}_{b1}=\frac{{\ddot{y}}_{b1}}{\gamma \omega^{2}},{\ddot{X}}_{b2}=\frac{{\ddot{x}}_{b2}}{\gamma \omega^{2}},{\ddot{Y}}_{b2}=\frac{{\ddot{y}}_{b2}}{\gamma \omega^{2}}\\ {\hat{\theta }}_{i}=\frac{\tau R_{i}}{R_{i}+R_{o}}+\frac{2\pi }{N_{b}}(i-1),{\hat{r}}_{\theta i}=X\cos{\hat{\theta }}_{i}+Y\sin{\hat{\theta }}_{i}-\gamma \end{array}\right.$$

The explicit physical justification for choosing these specific characteristic scales in Equation (12) is to capture the intrinsic geometric and kinematic boundary conditions of the high-speed turbocharger rotor system. Specifically, the rotor mass m, the structural unbalance eccentricity e, and the synchronous running frequency ω are selected as the characteristic mass, length, and time scales, respectively. This selection normalizes the cross-coupled non-linear equations, eliminating pure scale effects and ensuring that the simulated dimensionless parameters directly represent the severity of the structural fault regardless of absolute structural dimensions.

After nondimensionalization, the total contact forces of the left and right bearings are expressed as follows:

(13) $$\left\{\begin{array}{l} F_{xb1}=-\sum_{i=1}^{N_{b}}K_{bb1}{\left[(X_{b1}\cos{\hat{\theta }}_{i}+Y_{b1}\sin{\hat{\theta }}_{i})-\gamma \right]}_{+}^{1.5}\cos{\hat{\theta }}_{i}\\ F_{yb1}=-\sum_{i=1}^{N_{b}}K_{bb1}{\left[(X_{b1}\cos{\hat{\theta }}_{i}+Y_{b1}\sin{\hat{\theta }}_{i})-\gamma \right]}_{+}^{1.5}\sin{\hat{\theta }}_{i}\\ F_{xb2}=-\sum_{i=1}^{N_{b}}K_{bb2}{\left[(X_{b2}\cos{\hat{\theta }}_{i}+Y_{b2}\sin{\hat{\theta }}_{i})-\gamma \right]}_{+}^{1.5}\cos{\hat{\theta }}_{i}\\ F_{yb2}=-\sum_{i=1}^{N_{D}}K_{bb2}{\left[(X_{b2}\cos{\hat{\theta }}_{i}+Y_{b2}\sin{\hat{\theta }}_{i})-\gamma \right]}_{+}^{1.5}\sin{\hat{\theta }}_{i} \end{array}\right.$$

The governing equations of the system are nondimensionalized as follows:

(14) $$\left\{\begin{array}{l} {\ddot{X}}_{b1}+C_{b1}{\dot{X}}_{b1}+K(X_{b1}-X_{r})=F_{xb1}\\ {\ddot{Y}}_{b1}+C_{b1}{\dot{Y}}_{b1}+K(Y_{b1}-Y_{r})=F_{yb1}-G\\ {\ddot{X}}_{r}+C_{r}{\dot{X}}_{r}+K\frac{m_{b2}}{m_{r}}(X_{r}-X_{b2})+K\frac{m_{b1}}{m_{r}}(X_{r}-X_{b1})=E\cos\tau \\ {\ddot{Y}}_{r}+C_{r}{\dot{Y}}_{r}+K\frac{m_{b2}}{m_{r}}(Y_{r}-Y_{b2})+K\frac{m_{b1}}{m_{r}}(Y_{r}-Y_{b1})=E\cos\tau -G\\ {\ddot{X}}_{b2}+C_{b2}{\dot{X}}_{b2}+K(X_{b2}-X_{r})=F_{xb2}\\ {\ddot{Y}}_{b2}+C_{b2}{\dot{Y}}_{b2}+K(Y_{b2}-Y_{r})=F_{yb2}-G \end{array}\right.$$

Equation (14) represents the governing differential equations of motion of the healthy rolling bearing–rotor system.

### 2.2. Dynamic Modeling of a Faulty Rolling Bearing–Rotor System

#### 2.2.1. Rub-Impact Fault

In this study, the Coulomb friction model is adopted, and the friction coefficient is assumed to be constant and independent of the relative sliding velocity at the contact interface.

**Figure 3:**
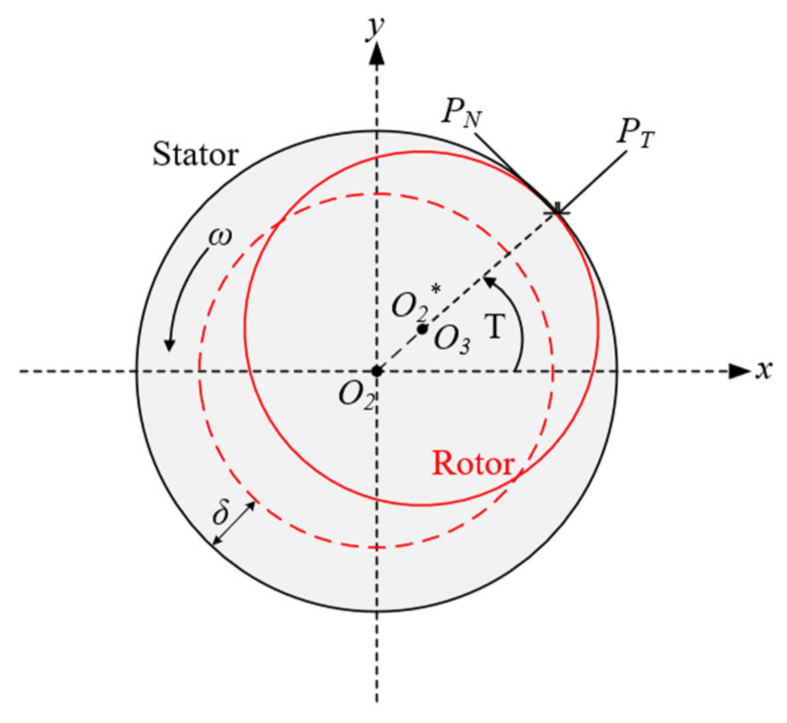
Schematic diagram of rubbing fault.

Figure 3 illustrates the rotor rub-impact mechanism: when local contact occurs between the outer surface of the rotor and the inner surface of the stator, a tangential friction force $P_{T}$ and a normal contact force $P_{N}$ are generated. Based on this, the rub-impact force model is established as follows:

(15) $$\left\{\begin{array}{l} r=O_{2}O_{2}^{*}=\sqrt{\left(x^{2}+y^{2}\right)}\\ P_{N}=\left\{\begin{array}{ll} 0 & ,(r<\delta )\\ (r-\delta )k_{c} & ,(r\geq \delta ) \end{array}\right.\\ P_{T}=fP_{N} \end{array}\right.$$

The tangential friction force and normal contact force induced by rub-impact are decomposed into the x- and y-directions as follows:

(16) PxPy=−(r−δ)kcr1−f f1xy,r≥δPx=Py=0,r<δ

Due to the inherent eccentricity between the rotor centroid and its geometric center, Figure 2 also represents the schematic of the rub-impact fault. The governing differential equations of motion of the rub-impact rotor system, established based on the Lagrange equations, are given in Equation (17).

(17) $$\left\{\begin{array}{l} m_{b1}{\ddot{x}}_{b1}+c_{b1}{\dot{x}}_{b1}+k(x_{b1}-x_{r})=f_{bx1}\\ m_{b1}{\ddot{y}}_{b1}+c_{b1}{\dot{y}}_{b1}+k(y_{b1}-y_{r})=f_{by1}-m_{b1}g\\ m_{r}{\ddot{x}}_{r}+c_{r}{\dot{x}}_{r}+k(x_{r}-x_{b1})+k(x_{r}-x_{b2})=m_{r}e\omega^{2}\cos(\omega t)+P_{x}\\ m_{r}{\ddot{y}}_{r}+c_{r}{\dot{y}}_{r}+k(y_{r}-y_{b1})+k(y_{r}-y_{b2})=m_{r}e\omega^{2}\sin(\omega t)+P_{y}-m_{r}g\\ m_{b2}{\ddot{x}}_{b2}+c_{b2}{\dot{x}}_{b2}+k(x_{b2}-x_{r})=f_{bx2}\\ m_{b2}{\ddot{y}}_{b2}+c_{b2}{\dot{y}}_{b2}+k(y_{b2}-y_{r})=f_{by2}-m_{b2}g \end{array}\right.$$

To facilitate numerical analysis and parameter normalization, a nondimensionalization procedure is introduced. The governing dynamic equations of the system are nondimensionalized using Equation (12), yielding:

(18) $$\left\{\begin{array}{l} {\ddot{X}}_{b1}+C_{b1}{\dot{X}}_{b1}+K(X_{b1}-X_{r})=F_{xb1}\\ {\ddot{Y}}_{b1}+C_{b1}{\dot{Y}}_{b1}+K(Y_{b1}-Y_{r})=F_{yb1}-G\\ {\ddot{X}}_{r}+C_{r}{\dot{X}}_{r}+K\frac{m_{b2}}{m_{r}}(X_{r}-X_{b2})+K\frac{m_{b1}}{m_{r}}(X_{r}-X_{b1})=Ecos\tau +p_{x}\\ {\ddot{Y}}_{r}+C_{r}{\dot{Y}}_{r}+K\frac{m_{b2}}{m_{r}}\left(Y_{r}-Y_{b2}\right)+K\frac{m_{b1}}{m_{r}}\left(Y_{r}-Y_{b1}\right)=Ecos\tau +p_{y}-G\\ {\ddot{X}}_{b2}+C_{b2}{\dot{X}}_{b2}+K(X_{b2}-X_{r})=F_{xb2}\\ {\ddot{Y}}_{b2}+C_{b2}{\dot{Y}}_{b2}+K(Y_{b2}-Y_{r})=F_{yb2}-G \end{array}\right.$$

Equation (18) represents the governing dynamic equations of the rotor system with a single rub-impact fault.

#### 2.2.2. Rub-Impact and Bearing Raceway Compound Faults

When local defects occur on the surface of bearing components due to material fatigue, periodic impact excitations with specific characteristic passing frequencies are generated. Taking inner-race defects as an example, such faults can induce a decaying vibratory response of the system during operation, significantly altering the normal vibration characteristics of the bearing. These fault characteristic frequencies are directly related to the structural parameters of the bearing and its operating conditions. A specific type of rolling bearing is selected as the object of analysis, and its detailed structural parameters are listed in Table 1.

Let the fault characteristic frequencies of the outer race, inner race, and rolling elements of the rolling bearing be denoted as $f_{o}$ and $f_{i}$, respectively, and let $f_{r}$ denote the rotational frequency of the rotor. The specific expressions for the fault characteristic frequencies are given in Table 2.

**Figure 4:**
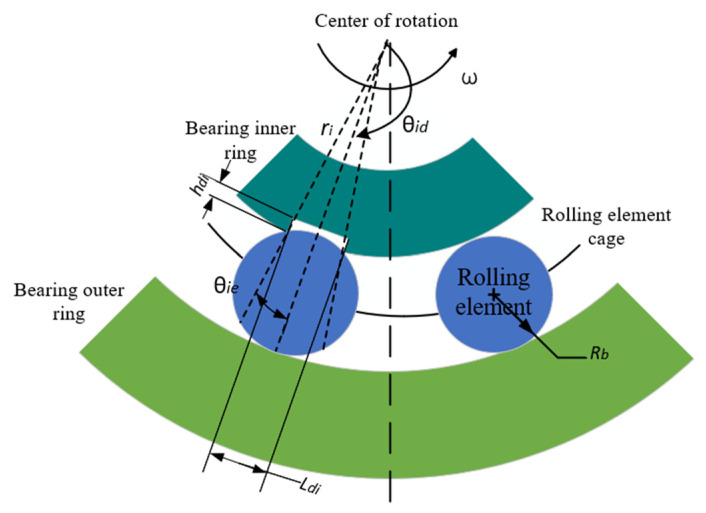
Schematic diagram of bearing inner ring fault.

As shown in Figure 4, when a localized defect exists on the inner race, the passage of rolling elements over the defect region induces impact vibrations and deformation. Since the inner race rotates synchronously with the rotor, the contact position between the rolling elements and the defect continuously varies with the rotation of the inner race, resulting in periodically fluctuating impact loads.

When the rolling element is located within the inner-race defect region, the geometric relationship among the defect half-angle $\theta_{ie}$, the defect width $L_{di}$, and the outer radius of the inner race $r_{i}$ is given by Equation (19):

(19) $$\theta_{\mathrm{ie}}=arcsin\left(\frac{L_{di}}{2r_{i}}\right)$$

When the rolling element enters the inner-race defect region, the angular position of the defect center varies with time due to the rotation of the inner-race, i.e., $\theta_{id}(t)=\theta_{id}(0)+\omega_{1}t$. Therefore, the inner race fault condition must satisfy Equation (20):

(20) $$abs\{mod(\varphi_{i}-\theta_{id}(t),2\pi )\}\leq \theta_{\mathrm{ie}}$$

The additional displacement induced by the localized defect on the inner race of the bearing is expressed as follows:

(21) $$h_{d}\left\{\begin{array}{l} \Delta h_{di}abs\{mod(\varphi_{i}-\theta_{id}(t),2\pi )\}\leq \theta_{\mathrm{ie}}\\ 0\;\mathrm{other} \end{array}\right.$$

If $\Delta h_{di}<h_{di}$, $\Delta h_{di}$ can be obtained from the geometric relationship as follows:

(22) $$\Delta h_{di}=r_{i}-\sqrt{r_{i}^{2}-{\left(\frac{L_{di}}{2}\right)}^{2}}$$

When a rolling element falls into the defect, the clearance is altered, which in turn leads to variations in the Hertzian contact force of the rolling bearing. In this section, it is assumed that the inner-race defect exists only in the left bearing. Accordingly, the resultant Hertzian contact forces of the bearing in the x- and y-directions can be expressed as follows:

(23) $$\left\{\begin{array}{l} f_{ixb1}=-\sum_{i=1}^{N_{b}}k_{bb1}{\left[(x_{b1}\cos\theta_{i}+y_{b1}\sin\theta_{i})-\gamma -\Delta h_{di}\right]}_{+}^{1.5}\cos\theta_{i}\\ f_{\mathrm{iy}b1}=-\sum_{i=1}^{N_{b}}k_{bb1}{\left[(x_{b1}\cos\theta_{i}+y_{b1}\sin\theta_{i})-\gamma -\Delta h_{di}\right]}_{+}^{1.5}\sin\theta_{i}\\ f_{xb2}=-\sum_{i=1}^{N_{b}}k_{bb2}{\left[(x_{b2}\cos\theta_{i}+y_{b2}\sin\theta_{i})-\gamma \right]}_{+}^{1.5}\cos\theta_{i}\\ f_{yb2}=-\sum_{i=1}^{N_{b}}k_{bb2}{\left[(x_{b2}\cos\theta_{i}+y_{b2}\sin\theta_{i})-\gamma \right]}_{+}^{1.5}\sin\theta_{i} \end{array}\right.$$

Therefore, based on Equation (11), the inner-race defect is incorporated, and the governing dynamic equations of the rolling bearing–rotor system can be expressed as follows:

(24) $$\left\{\begin{array}{l} m_{b1}{\ddot{x}}_{b1}+c_{b1}{\dot{x}}_{b1}+k(x_{b1}-x_{r})=f_{ibx1}\\ m_{b1}{\ddot{y}}_{b1}+c_{b1}{\dot{y}}_{b1}+k(y_{b1}-y_{r})=f_{iby1}-m_{b1}g\\ m_{r}{\ddot{x}}_{r}+c_{r}{\dot{x}}_{r}+k(x_{r}-x_{b1})+k(x_{r}-x_{b2})=m_{r}e\omega^{2}\cos(\omega t)\\ m_{r}{\ddot{y}}_{r}+c_{r}{\dot{y}}_{r}+k(y_{r}-y_{b1})+k(y_{r}-y_{b2})=m_{r}e\omega^{2}\sin(\omega t)-m_{r}g\\ m_{b2}{\ddot{x}}_{b2}+c_{b2}{\dot{x}}_{b2}+k(x_{b2}-x_{r})=f_{bx2}\\ m_{b2}{\ddot{y}}_{b2}+c_{b2}{\dot{y}}_{b2}+k(y_{b2}-y_{r})=f_{by2}-m_{b2}g \end{array}\right.$$

To facilitate numerical analysis and parameter normalization, the governing dynamic equations of the system are nondimensionalized using Equation (12). After nondimensionalization, the total contact forces of the left and right bearings are expressed as follows:

(25) $$\left\{\begin{array}{l} F_{ixb1}=-\sum_{i=1}^{N_{b}}K_{bb1}{\left[(X_{b1}\cos{\hat{\theta }}_{i}+Y_{b1}\sin{\hat{\theta }}_{i})-\gamma -\Delta h_{di}\right]}_{+}^{1.5}\cos{\hat{\theta }}_{i}\\ F_{\mathrm{iy}b1}=-\sum_{i=1}^{N_{b}}K_{bb1}{\left[(X_{b1}\cos{\hat{\theta }}_{i}+Y_{b1}\sin{\hat{\theta }}_{i})-\gamma -\Delta h_{di}\right]}_{+}^{1.5}\sin{\hat{\theta }}_{i}\\ F_{xb2}=-\sum_{i=1}^{N_{b}}K_{bb2}{\left[(X_{b2}\cos{\hat{\theta }}_{i}+Y_{b2}\sin{\hat{\theta }}_{i})-\gamma \right]}_{+}^{1.5}\cos{\hat{\theta }}_{i}\\ F_{yb2}=-\sum_{i=1}^{N_{b}}K_{bb2}{\left[(X_{b2}\cos{\hat{\theta }}_{i}+Y_{b2}\sin{\hat{\theta }}_{i})-\gamma \right]}_{+}^{1.5}\sin{\hat{\theta }}_{i} \end{array}\right.$$

After nondimensional substitution, Equation (24) is transformed into the following form:

(26) $$\left\{\begin{array}{l} {\ddot{X}}_{b1}+C_{b1}{\dot{X}}_{b1}+K(X_{b1}-X_{r})=F_{ixb1}\\ {\ddot{Y}}_{b1}+C_{b1}{\dot{Y}}_{b1}+K(Y_{b1}-Y_{r})=F_{iyb1}-G\\ {\ddot{X}}_{r}+C_{r}{\dot{X}}_{r}+K\frac{m_{b2}}{m_{r}}(X_{r}-X_{b2})+K\frac{m_{b1}}{m_{r}}(X_{r}-X_{b1})=E\cos\tau \\ {\ddot{Y}}_{r}+C_{r}{\dot{Y}}_{r}+K\frac{m_{b2}}{m_{r}}(Y_{r}-Y_{b2})+K\frac{m_{b1}}{m_{r}}(Y_{r}-Y_{b1})=Ecos\tau +p_{y}-G\\ {\ddot{X}}_{b2}+C_{b2}{\dot{X}}_{b2}+K(X_{b2}-X_{r})=F_{xb2}\\ {\ddot{Y}}_{b2}+C_{b2}{\dot{Y}}_{b2}+K(Y_{b2}-Y_{r})=F_{yb2}-G \end{array}\right.$$

Equation (26) represents the governing dynamic equations of the rotor system with an inner-race fault.

Based on the dynamic model of the healthy rolling bearing–rotor system, the rub-impact force at the disk location and the inner-race defect at the left bearing are introduced. By combining Equations (17) and (24), the governing equations of the coupled rub-impact and inner-race fault system are established. After nondimensionalization using Equation (12), the resulting expressions are obtained as follows:

(27) $$\left\{\begin{array}{l} {\ddot{X}}_{b1}+C_{b1}{\dot{X}}_{b1}+K(X_{b1}-X_{r})=F_{ixb1}\\ {\ddot{Y}}_{b1}+C_{b1}{\dot{Y}}_{b1}+K(Y_{b1}-Y_{r})=F_{iyb1}-G\\ {\ddot{X}}_{r}+C_{r}{\dot{X}}_{r}+K\frac{m_{b2}}{m_{r}}(X_{r}-X_{b2})+K\frac{m_{b1}}{m_{r}}(X_{r}-X_{b1})=E\cos\tau +p_{x}\\ {\ddot{Y}}_{r}+C_{r}{\dot{Y}}_{r}+K\frac{m_{b2}}{m_{r}}\left(Y_{r}-Y_{b2}\right)+K\frac{m_{b1}}{m_{r}}\left(Y_{r}-Y_{b1}\right)=Ecos\tau +p_{y}-G\\ {\ddot{X}}_{b2}+C_{b2}{\dot{X}}_{b2}+K(X_{b2}-X_{r})=F_{xb2}\\ {\ddot{Y}}_{b2}+C_{b2}{\dot{Y}}_{b2}+K(Y_{b2}-Y_{r})=F_{yb2}-G \end{array}\right.$$

The above equations represent the governing dynamic equations of the coupled rub-impact and inner-race fault system.

**Figure 5:**
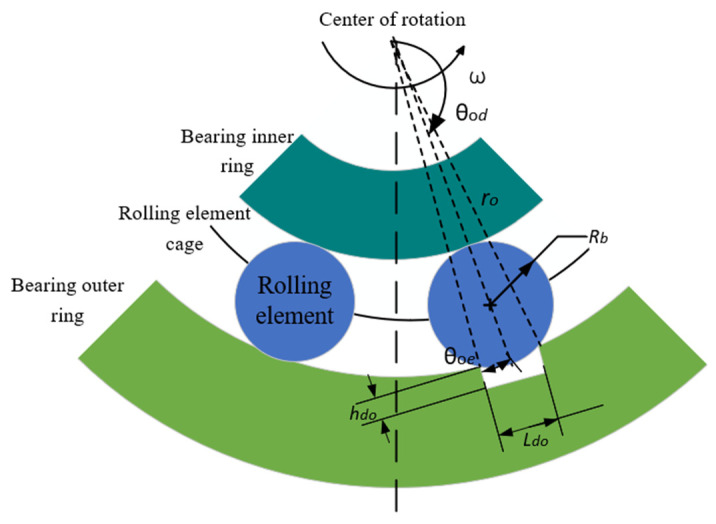
Schematic diagram of outer ring bearing fault.

Figure 5 illustrates the schematic diagram of the bearing outer-race fault.

The modeling procedure of the outer-race fault is similar to that of the inner-race fault described above. The primary difference lies in the definition of the fault location and the corresponding angular variables. Therefore, for brevity, the detailed derivation process is omitted, and only the nondimensionalized dynamic equations are directly presented. Accordingly, the governing dynamic equations of the outer-race fault system can be expressed as follows:

(28) $$\left\{\begin{array}{l} {\ddot{X}}_{b1}+C_{b1}{\dot{X}}_{b1}+K(X_{b1}-X_{r})=F_{oxb1}\\ {\ddot{Y}}_{b1}+C_{b1}{\dot{Y}}_{b1}+K(Y_{b1}-Y_{r})=F_{oyb1}-G\\ {\ddot{X}}_{r}+C_{r}{\dot{X}}_{r}+K\frac{m_{b2}}{m_{r}}(X_{r}-X_{b2})+K\frac{m_{b1}}{m_{r}}(X_{r}-X_{b1})=E\cos\tau +p_{x}\\ {\ddot{Y}}_{r}+C_{r}{\dot{Y}}_{r}+K\frac{m_{b2}}{m_{r}}(Y_{r}-Y_{b2})+K\frac{m_{b1}}{m_{r}}(Y_{r}-Y_{b1})=Ecos\tau +p_{y}-G\\ {\ddot{X}}_{b2}+C_{b2}{\dot{X}}_{b2}+K(X_{b2}-X_{r})=F_{xb2}\\ {\ddot{Y}}_{b2}+C_{b2}{\dot{Y}}_{b2}+K(Y_{b2}-Y_{r})=F_{yb2}-G \end{array}\right.$$

The simplification of the rotor–bearing system into a reduced-order, lumped-mass Jeffcott model is quantitatively and physically justified based on established benchmarks in the rotor dynamics literature. For a symmetric layout where a rigid disk is centrally mounted and the operating speed remains well below the sub-critical thresholds, prior experimental and numerical studies have rigorously verified the high fidelity of such simplified parameters. Specifically, Alsaleh et al. [35] demonstrated that a two-degrees-of-freedom Jeffcott model can predict lateral flexural vibrations with a quantitative error of less than 5% compared to real rotor kit measurements under rigid conditions. Furthermore, as investigated by Patel and Darpe [36] in multi-fault rotor dynamics, the continuous shaft mass and gyroscopic coupling exert a negligible influence on the primary transient trajectories and rub-impact frequency modulations under these operational boundaries. Consequently, the adopted model retains a high level of qualitative and quantitative accuracy for capturing the non-linear compound fault features while maintaining computational efficiency.

## 3. Dynamic Characteristics Analysis of the Faulty Rolling Bearing–Rotor System

### 3.1. Dynamic Characteristics Analysis of the Rotor System with a Single Rub-Impact Faul

**Figure 6:**
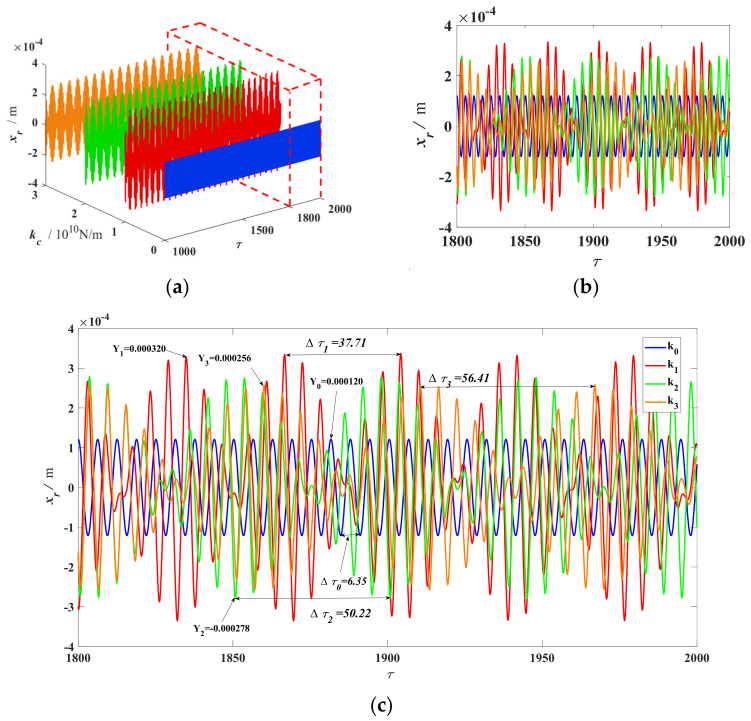
Time histories of disk vibration in the x-direction under rotor–stator rub-impact at different stator stiffness values (where $k_{0}$:$k_{c}$ = 0 N/m, $k_{1}$:$k_{c}$ = 1 × 10^10^ N/m, $k_{2}$:$k_{c}$ = 2 × 10^10^ N/m, and $k_{3}$:$k_{c}$ = 3 × 10^10^ N/m). (**a**) the time history of disk vibration in the x-direction under different stator stiffness conditions during rub-impact between the disk and the stator. (**b**) a locally enlarged view (**c**) further magnifies Figure 6b.

The study object in this section is the rotor disk. Figure 6a presents the time history of disk vibration in the x-direction under different stator stiffness conditions during rub-impact between the disk and the stator. When the rub-impact stiffness $k_{0}$ = 0, i.e., no rub-impact fault occurs, the time-domain response exhibits a smooth and continuous sinusoidal waveform with a stable peak amplitude of $Y_{0}$ = 0.000120 m and a regular dimensionless time interval of ${\Delta \tau }_{0}$ = 6.35. The other three curves in Figure 6a correspond to the dynamic responses under different stiffness values. Figure 6b shows a locally enlarged view, and Figure 6c further magnifies Figure 6b, illustrating that the non-linear characteristics are significantly enhanced and the response deviates from a regular sinusoidal pattern. Quantitatively, as labeled in the figures, under the rub-impact condition of $k_{1}$, the peak vibration amplitude sharply amplifies to $Y_{1}$ = 0.000320 m, representing a 167% increase relative to the healthy baseline $Y_{0}$. Concurrently, the system transitions into modulated waveforms where the characteristic dimensionless time intervals and periods are precisely captured as ${\Delta \tau }_{1}$ = 37.71$, $Y_{2}$ = −0.000278 m, ${\Delta \tau }_{2}$ = 50.22$, $Y_{3}$ = 0.000256 m, and ${\Delta \tau }_{3}$ = 56.41.

**Figure 7:**
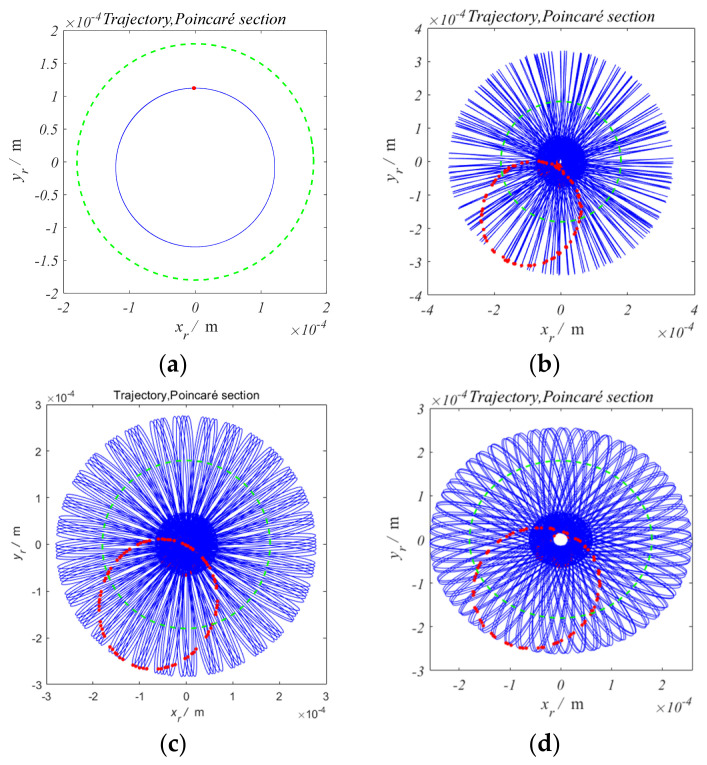
Poincaré sections and shaft center orbits of the disk under different rub-impact stiffness conditions. (**a**) $k_{0}=0$; (**b**) $k_{1}=1\times 10^{10}\;N/m$; (**c**) $k_{2}=2\times 10^{10}\;N/m$; (**d**) $k_{3}=3\times 10^{10}\;N/m$.

Figure 7 presents the Poincaré sections and shaft center orbits of the rotor disk under different rub-impact stiffness conditions. In Figure 7a, corresponding to the non-rub condition, the shaft center orbit exhibits a regular elliptical shape and does not cross the rub-impact boundary. Figure 7b shows that the shaft center orbit presents a typical flower-like pattern, with uniformly distributed and relatively smooth petals, indicating mild friction. The orbit range is significantly enlarged and crosses the rub-impact boundary. In Figure 7c,d, as the rub-impact stiffness increases, the non-uniform contact force leads to partial deformation of the petals. The petal tips become sharper and the impact force becomes more concentrated, exhibiting pronounced non-linear impact characteristics and indicating the possible occurrence of local stress concentration.

**Figure 8:**
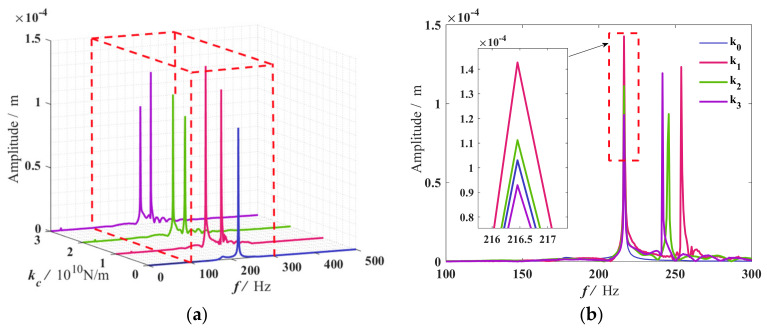
Frequency spectra of the disk vibration in the x-direction under different rub-impact stiffness conditions (where $k_{0}$:$k_{c}$ = 0 N/m, $k_{1}$:$k_{c}$ = 1 × 10^10^ N/m, $k_{2}$:$k_{c}$ = 2 × 10^10^ N/m, and $k_{3}$:$k_{c}$ = 3 × 10^10^ N/m). (**a**) full spectrum comparison; (**b**) detailed view.

Figure 8 presents the frequency spectra of the rotor disk in the x-direction under different rub-impact stiffness conditions. As shown in Figure 8a, under the non-rub condition, only the fundamental frequency component is present. Figure 8b provides a locally enlarged view of the region highlighted in Figure 8a. It can be observed that sideband frequency components emerge in the spectrum under different rub-impact stiffness conditions, and subharmonic components gradually appear as the rub-impact intensity increases. The results indicate a significant correlation between the stator stiffness and the rub-impact severity. This significant reduction in the fundamental frequency amplitude under increasing stiffness indicates that the stator stiffness has a suppressive effect on rotor unbalance vibration. This behavior is primarily governed by a structural over-constraint stiffening effect combined with non-linear spectral energy redistribution. When the stator rubbing stiffness amplifies, the boundary contact acts as a harsh, non-linear parallel support constraining the lateral displacement of the rotor disk. This contact interaction introduces a significant structural stiffening constraint that shifts the system’s instantaneous natural frequencies away from the synchronous running frequency, thereby suppressing the resonant unbalance response at the fundamental frequency. Concurrently, from the perspective of energy conservation, the severe intermittent collision forces act as a strong periodic modulation, transferring a massive portion of the total vibrational energy from the primary fundamental component into higher-order harmonics and fractional sidebands.

**Figure 9:**
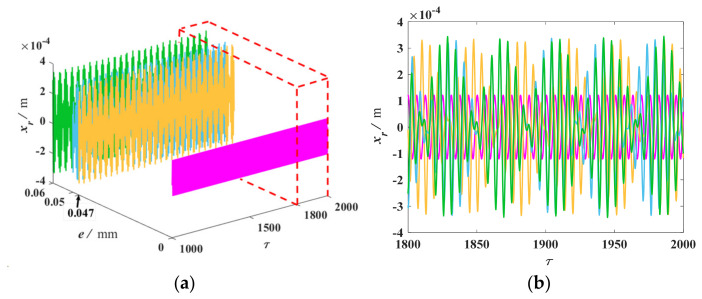

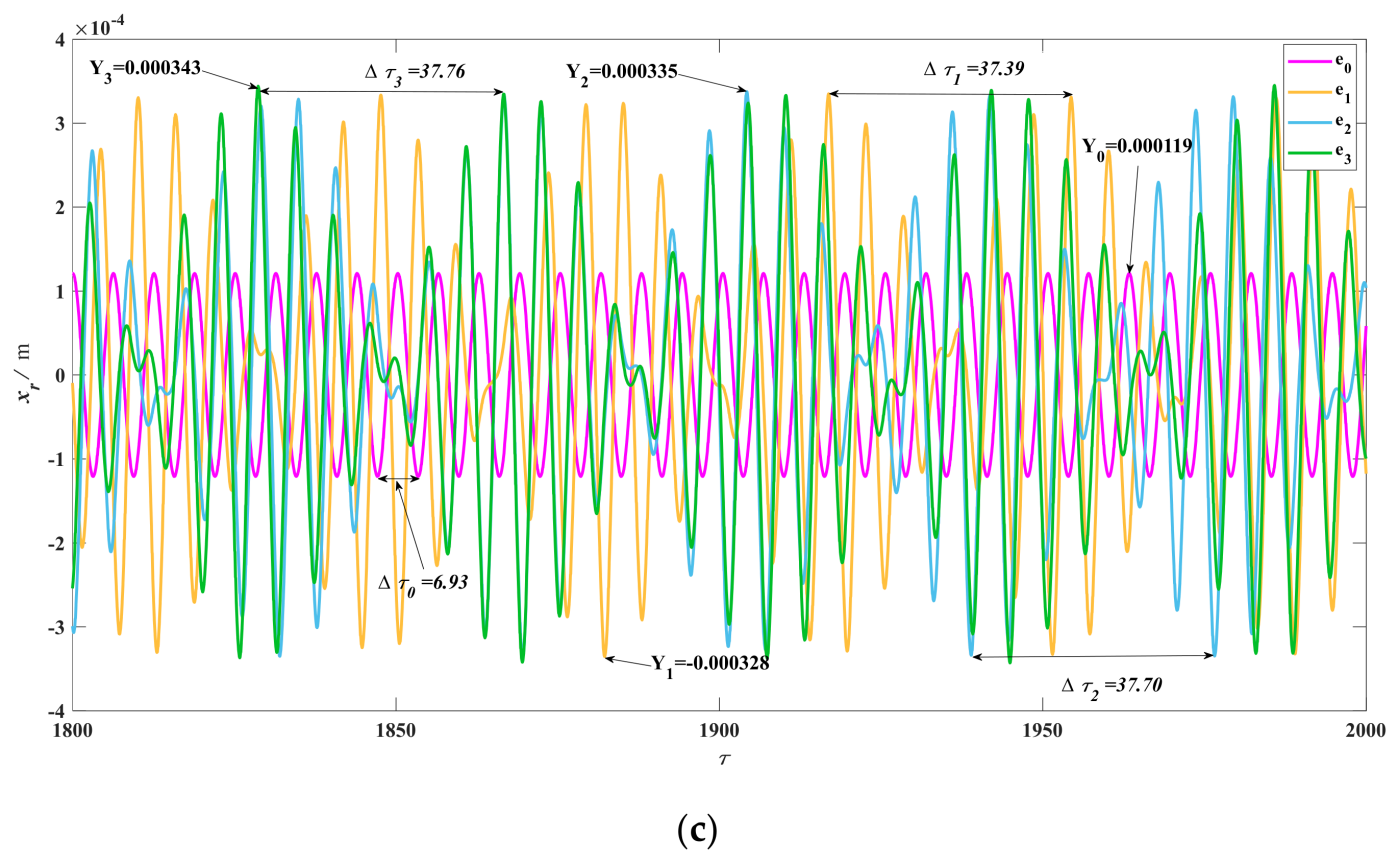
Time-domain vibration response histories of the disk under different eccentricities (where $e_{0}$:e=0 mm, $e_{1}$:e=0.047 mm, $e_{2}$:e=0.05 mm, and $e_{3}$:e=0.06 mm). (**a**) the dynamic responses in the x-direction under rub-impact conditions with different eccentricities; (**b**) shows a locally enlarged view; (**c**) further magnifies Figure 9b.

In Figure 9a, the curves represent the time-domain responses of the rotor disk vibration under different eccentricity values. The case of $e_{0}$ corresponds to the non-rub condition, in which the vibration response exhibits a smooth and regular sinusoidal waveform with a stable peak amplitude of 0.000119 m and a regular dimensionless time interval of 6.93. The other three curves in Figure 9a correspond to the dynamic responses in the x-direction under rub-impact conditions with different eccentricities. Figure 9b shows a locally enlarged view, and Figure 9c further magnifies Figure 9b. A comparison of the results in Figure 9c indicates that the non-linear characteristics of the system are significantly enhanced. The time-domain response deviates from a regular sinusoidal waveform and becomes irregular. Quantitatively, as labeled in the figures, under the rub-impact condition of $e_{1}$, the peak vibration amplitude reaches −0.000328 m, and the characteristic dimensionless time interval is precisely captured as 37.39. For the cases of $e_{2}$ and $e_{3}$, the corresponding dynamic indicators further transition, where the distinct peak values are extracted as 0.000335 m and 0.000343 m, respectively, while the remaining critical dimensionless time periods are quantitatively defined as 37.70 and 37.76.

**Figure 10:**
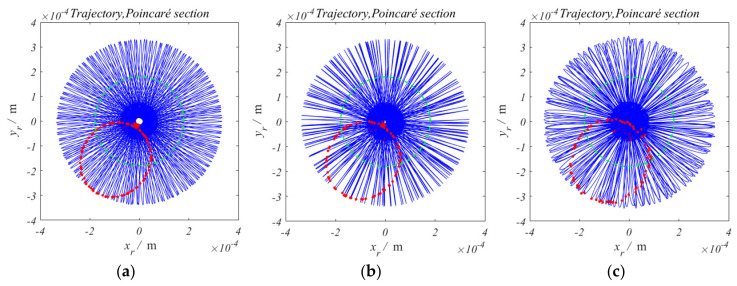
Poincaré sections and shaft center trajectories of the disk under different eccentricities. (**a**) $e_{1}=0.047\mathrm{mm}$; (**b**) $e_{2}=0.05\mathrm{mm}$; (**c**) $e_{3}=0.06\mathrm{mm}$.

Figure 10a–c present the Poincaré sections and shaft center orbits of the rotor system under different eccentric masses. A comparison of the dynamic responses under various eccentricity levels indicates that the shaft center orbit consistently exhibits typical rub-impact characteristics as the eccentricity increases; however, its morphology changes with increasing eccentricity. At small eccentricity, intermittent rub-impact occurs, and the orbit presents a hollow flower-like pattern. The sharp petal tips correspond to instantaneous contact, while the central region remains empty due to non-contact. As the eccentricity increases, the rotor is continuously biased toward the stator side, resulting in an extended contact duration and a gradual transition to full annular friction. Consequently, the central region of the orbit becomes densely populated with data points.

**Figure 11:**
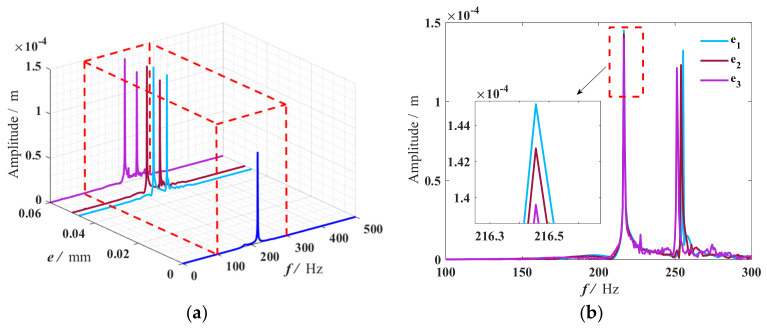
Comparative spectra of the disk in the x-direction under different eccentricities (where $e_{1}$:e=0.047 mm, $e_{2}$:e=0.05 mm, and $e_{3}$:e=0.06 mm). (**a**) full spectrum comparison; (**b**) detailed view.

Figure 11a,b present a comparison of the frequency spectra of the rotor disk in the x-direction under different eccentricity levels within the same coordinate system. The spectral analysis indicates that the amplitude of the fundamental synchronous harmonic decreases with increasing eccentricity. It is worth noting that this trend, when contrasted with the simultaneous amplification of time-domain peak trajectories, does not imply a physical contradiction. Rather, it represents a classic non-linear energy redistribution and interface friction dissipation trait governed by the global energy balance. As the eccentricity progresses, the severe, non-continuous stator contacts act as a strong frequency mixer, forcefully modulating the macro-synchronous energy originally concentrated at the fundamental frequency into high-frequency grazing sidebands, discrete multipliers, and broadband continuous spectral components. Concurrently, the heightened friction work and collision damping at the rubbing boundaries serve as an energy sink, directly dampening the single-frequency structural recovery of the shaft. Consequently, an increase in eccentricity intensifies the global unbalance excitation and severely aggravates the multi-frequency non-linear vibrations induced by rub-impact. In summary, for fault systems involving rotor dynamics, proper control of eccentricity plays a critical role in suppressing broadband shock propagation and maintaining global operational stability.

### 3.2. Dynamic Characteristics Analysis of the Coupled Rub-Impact and Inner-Race Fault System

**Figure 12:**
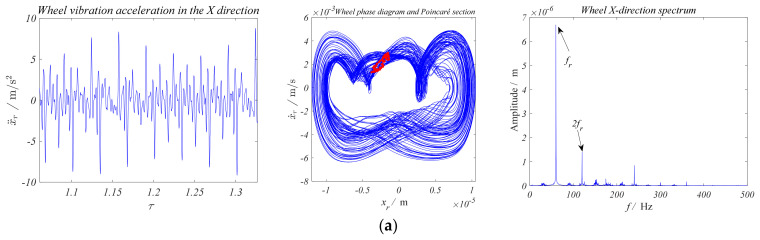

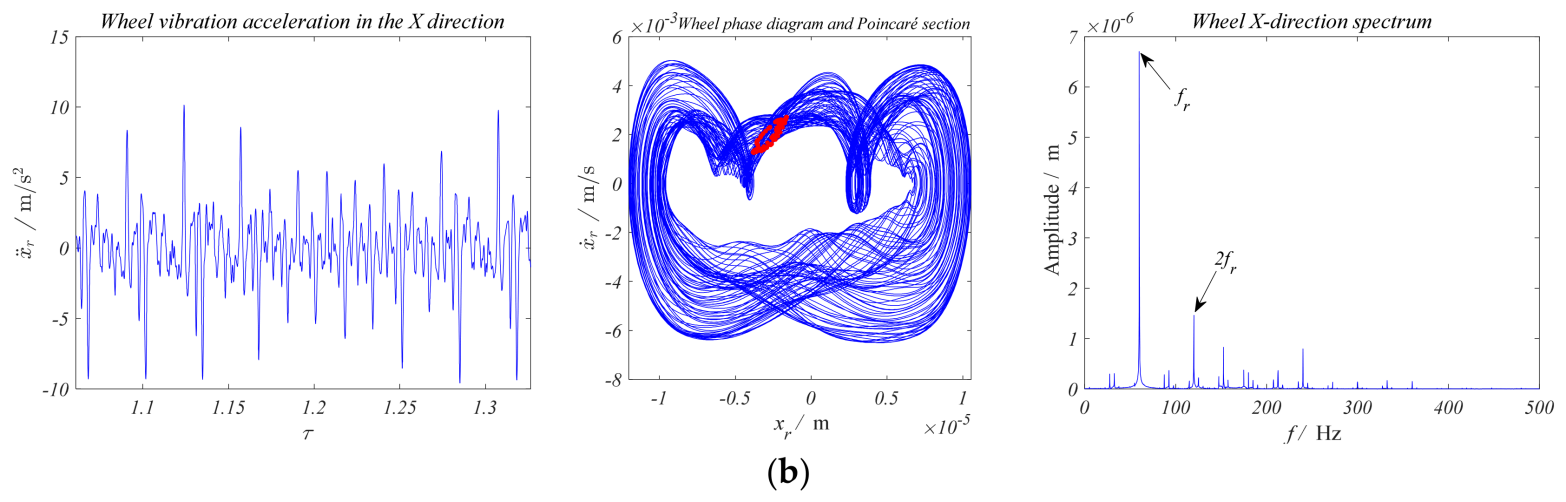
Comparison of the dynamic characteristics of the disk under rubbing fault and rubbing–bearing inner-race compound fault conditions. (**a**) under pure rubbing fault condition; (**b**) under rubbing–bearing fault conditions.

With a 2 mm inner-race defect width as the sole operational variant, the dynamic responses of the disk are compared under pure rubbing (Figure 12a) and rubbing–bearing compound fault conditions (Figure 12b). The comparison reveals that the non-linear behavior of the disk is heavily dominated by the rubbing fault. Specifically, the frequency spectra are characterized by the rotational frequency and its high-order harmonics, while the phase trajectories manifest as distorted, complex patterns with dense geometric superpositions.

**Figure 13:**
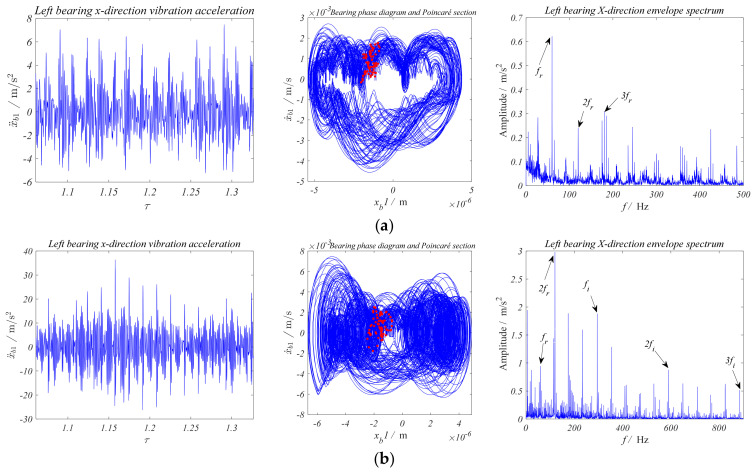
Comparison of dynamic characteristics between the left bearing-end rubbing fault and the rubbing–bearing inner-race composite fault system. (**a**) under pure rubbing fault; (**b**) under rubbing–inner-race compound faults.

With a 2 mm inner-race defect width as the sole operational variant, the dynamic responses of the left bearing are compared under pure rubbing (Figure 13a) and rubbing–inner-race compound faults (Figure 13b). The comparison reveals that the vibration response at the bearing end exhibits a distinct superposition of both fault mechanisms. Specifically, the time-domain waveform demonstrates a combined shock profile, the phase trajectory becomes increasingly disordered, and the envelope spectrum simultaneously captures the rotational frequency, the inner-race fault characteristic frequency, and their modulation sidebands—closely matching the typical diagnostic traits of an inner-race defect.

#### 3.2.1. Analysis of the Influence of Rubbing Stiffness Variation on Dynamic Characteristics

**Figure 14:**
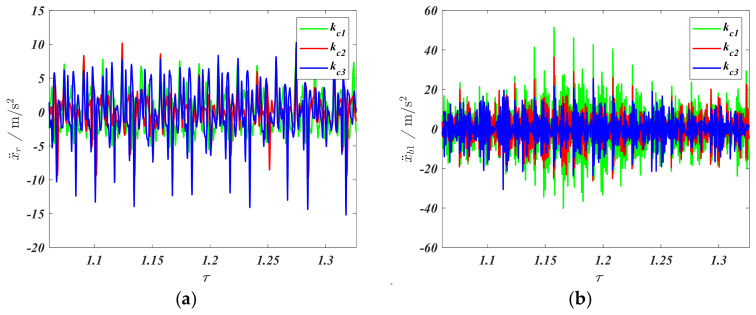
Time-history response of vibration acceleration of the system with varying rub-impact stiffness (where $k_{c1}$:$k_{c}$ = 1 × 10^10^ N/m, $k_{c2}$:$k_{c}$ = 2 × 10^10^ N/m, and $k_{c3}$:$k_{c}$ = 3 × 10^10^ N/m). (**a**) the disk;. (**b**) the left bearing.

To investigate the explicit influence of rub-impact stiffness on the dynamic characteristics of the compound fault system, Figure 14a,b present the x-direction vibration accelerations of the disk and left bearing, respectively, under increasing rub-impact stiffness. The time-domain waveforms show that higher stiffness causes the disk vibration to evolve from a slightly distorted sinusoidal wave into sustained, high-amplitude impact segments. Conversely, at the bearing end, the coupling between the transmitted rub-impact force and the inner-race fault increases waveform complexity but reduces the overall amplitude. This amplitude reduction is primarily attributed to the rub-impact force transitioning from intermittent impacts to continuous interface friction under high-stiffness constraints.

**Figure 15:**
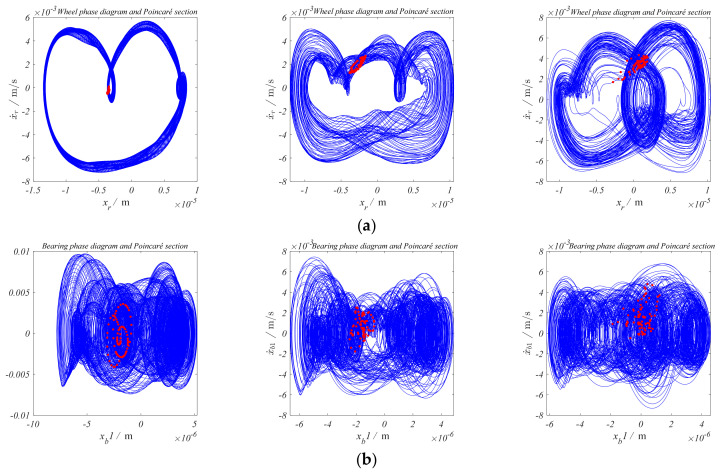
Phase portraits and Poincaré sections of the system with increasing rub-impact stiffness. (**a**) the phase portraits and Poincaré sections of the disk under increasing rub-impact stiffness; (**b**) the phase portraits and Poincaré sections of the left bearing under increasing rub-impact stiffness.

To evaluate the structural geometric evolution under varying boundary constraints, Figure 15a,b display the phase portraits and Poincaré sections of the disk and left bearing, respectively, under increasing rub-impact stiffness. Comparative analysis indicates that the phase trajectories of the disk are highly sensitive to the worsening rub-impact, exhibiting broadened orbits, multi-loop nesting, and an expanded phase space area that reflects an increasingly chaotic dynamical structure. In contrast, the phase portraits of the bearing end remain remarkably robust against the increasing stiffness, showing only marginal changes.

**Figure 16:**
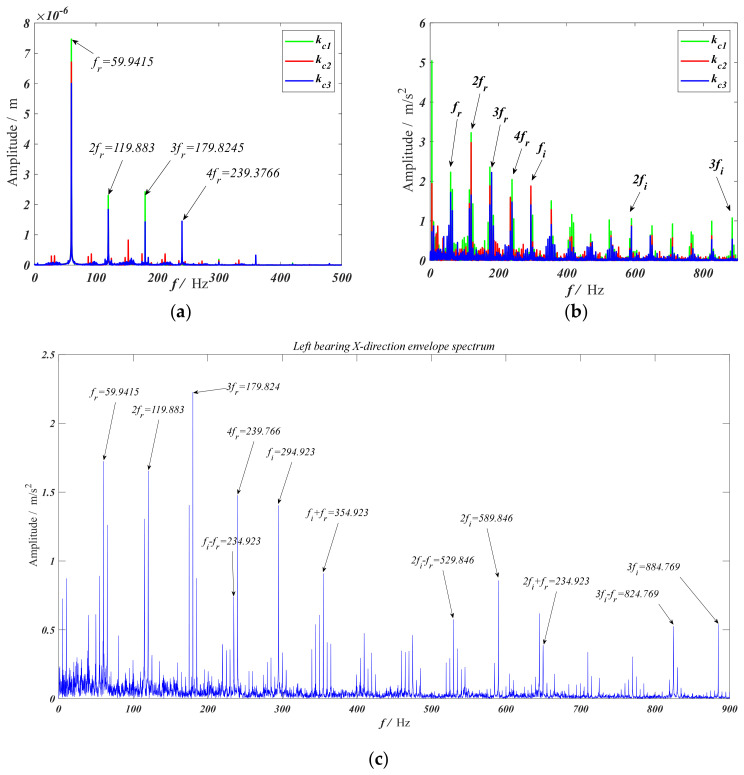
Frequency spectra and envelope spectra of the system with increasing rub-impact stiffness (where $k_{c1}$:$k_{c}$ = 1 × 10^10^ N/m, $k_{c2}$:$k_{c}$ = 2 × 10^10^ N/m, and $k_{c3}$:$k_{c}$ = 3 × 10^10^ N/m). (**a**) the x-direction frequency spectrum of the disk under increasing rub-impact stiffness; (**b**) envelope spectra of the left bearing under increasing rub-impact stiffness; (**c**) direct spectra of the left bearing.

Figure 16a–c display the x-direction frequency spectrum of the disk, alongside the envelope and direct spectra of the left bearing under increasing rub-impact stiffness. Here, $f_{r}$ and $f_{i}$ represent the fundamental rotational frequency and the inner-race fault characteristic frequency, respectively. The direct spectra exhibit prominent rotational frequency harmonics, while the bearing envelope spectrum clearly captures $f_{r}$, $f_{i}$, and their modulation sidebands. Overall, increasing stiffness intensifies the dynamic responses of both components through distinct mechanisms: the disk response is directly dictated by rub-impact severity, whereas the bearing response is jointly driven by transmitted impact forces and inner-race defects, yielding more intricate non-linear behavior.

#### 3.2.2. Dynamic Analysis of the Influence of Fault Width Variation on System Behavior

**Figure 17:**
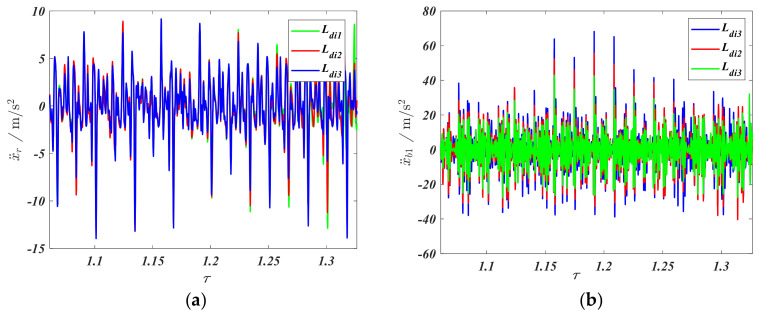
Time-domain vibration acceleration of the system with increasing bearing inner-race defect width in the x-direction (where $L_{di1}$:$L_{di}$ = 3 mm, $L_{di2}$:$L_{di}$ = 4 mm, and $L_{di3}$:$L_{di}$ = 5 mm). (**a**) the disk; (**b**) the left bearing.

**Figure 18:**
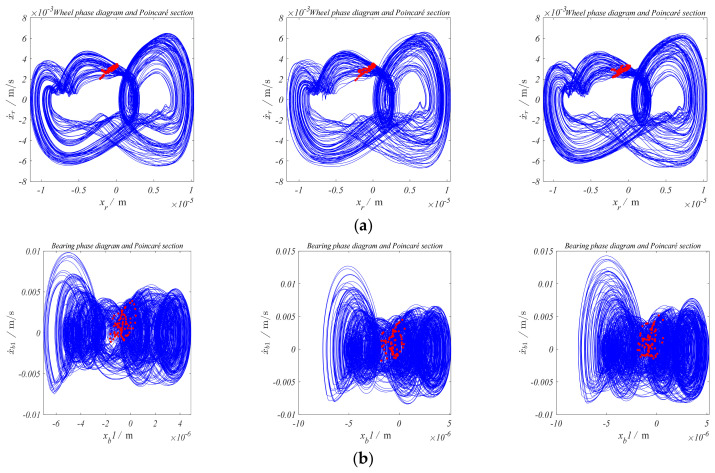
Phase portraits and Poincaré sections of the system with increasing bearing inner-race defect width. (**a**) the x-direction time-domain vibration accelerations of the disk under increasing inner-race defect width; (**b**) the x-direction time-domain vibration accelerations of the left bearing under increasing inner-race defect width.

**Figure 19:**
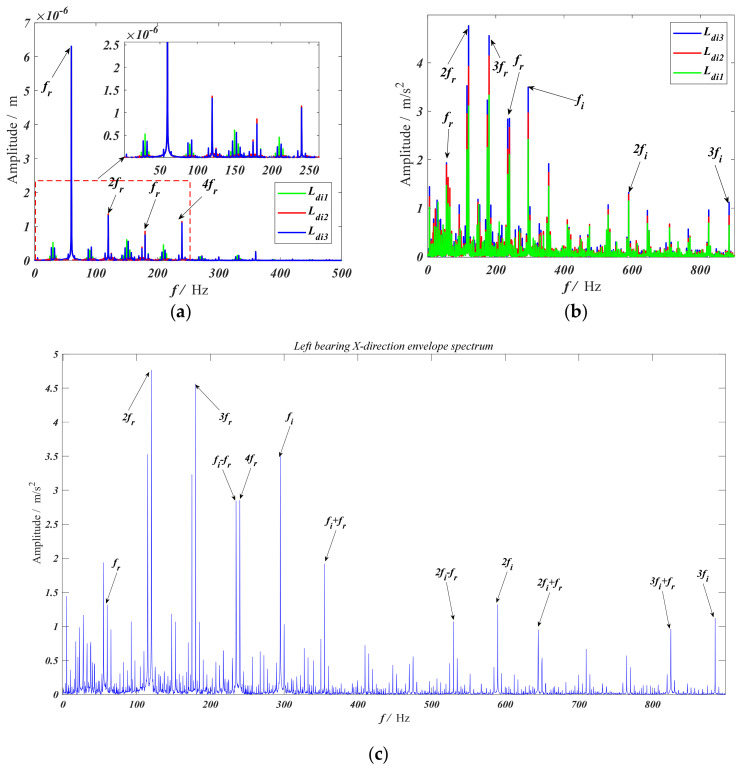
Frequency spectra and envelope spectra of the system with increasing bearing inner race defect width in the x-direction (where $L_{di1}$:$L_{di}$ = 3 mm, $L_{di2}$:$L_{di}$ = 4 mm, and $L_{di3}$:$L_{di}$ = 5 mm). (**a**) the x-direction frequency spectrum of the disk under increasing inner-race defect width; (**b**) the x-direction envelope of the left bearing under increasing inner-race defect width; (**c**) the x-direction direct spectra of the left bearing.

Figure 17, Figure 18 and Figure 19 present the system’s dynamic responses at a constant rotational speed under fixed eccentricity and clearance parameters, with varying bearing inner-race fault widths ($L_{di}$). Comparing these operational states effectively reveals the evolutionary characteristics and underlying mechanisms of the system dynamics induced by inner-race fault expansion.

Figure 18a,b present the x-direction time-domain vibration accelerations of the disk and left bearing, respectively, under increasing inner-race defect width. The time-domain analysis shows that as the fault width expands, the disk waveform remains dominated by sustained, high-amplitude rub-impact forces, maintaining a stable overall profile. Conversely, the bearing waveform amplitude increases significantly, reflecting a more pronounced superposition effect of the rub-impact excitation.

Figure 18a,b present the phase portraits and Poincaré sections of the disk and left bearing, respectively, under increasing inner-race defect width. The Poincaré sections show that the disk-end point set—already diffused by rub-impact—expands only marginally with increasing defect width, maintaining its structural morphology. In contrast, the bearing-end point set rapidly evolves from a compact cluster into a loosely dispersed, cloud-like distribution, signaling a marked increase in dynamical complexity. The phase trajectories corroborate this divergence: the bearing morphology is primarily sensitive to the inner-race defect, whereas the disk remains dominated by rub-impact. Notably, the disk’s trajectory area expands as the fault width increases, likely due to enhanced impact energy transmitted from the inner race.

Figure 19a–c display the x-direction frequency spectrum of the disk, alongside the envelope and direct spectra of the left bearing under increasing inner-race defect width. The spectral analysis shows that while the disk-end response exhibits minimal variations, the bearing end displays pronounced sensitivity; the amplitudes of the inner-race fault characteristic frequency ($f_{i}$) and its harmonics increase sharply, emerging as dominant spectral components. Overall, expanding defect width is first and most significantly captured by the bearing end’s diagnostic indicators. Conversely, the disk response remains heavily dominated by rub-impact severity, which largely masks the inner-race fault signatures until the defect becomes extreme.

### 3.3. Dynamic Analysis of the Rub-Impact–Outer-Race Compound Fault System

**Figure 20:**
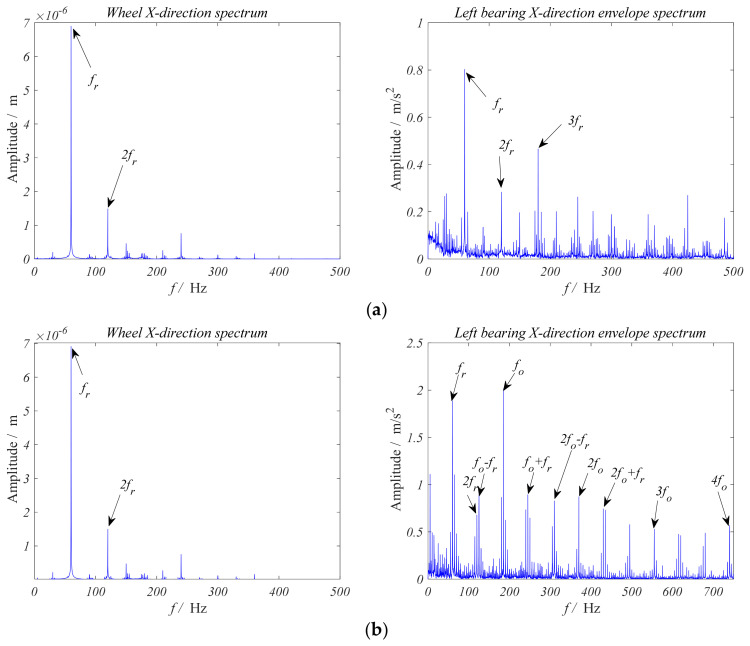
Comparison of the spectrum and envelope spectrum between the rubbing fault system and the rubbing–outer-race compound fault system. (**a**) frequency spectra and envelope spectra of the system with a single rub-impact fault in the x-direction. (**b**) frequency spectra and envelope spectra of the system with rub-impact–outer-race compound faults in the x-direction.

Figure 20 compares the x-direction direct and envelope spectra between the pure rubbing and rubbing–outer-race compound fault systems. Utilizing identical operational parameters as the previous chapter, numerical simulations demonstrate that the non-linear response at the disk remains heavily dominated by the rubbing fault, confirming that bearing-side defects exert a negligible macro-influence on disk dynamics. Under both outer- and inner-race fault conditions, the bearing-end responses exhibit consistent coupling traits, yielding highly similar time-domain waveforms and phase trajectories. Their primary divergence lies in the envelope spectra, which distinctly capture their respective fault characteristic frequencies and modulation sidebands.

#### 3.3.1. Dynamic Analysis of the Influence of Rub-Impact Stiffness Variation on System Behavior

**Figure 21:**
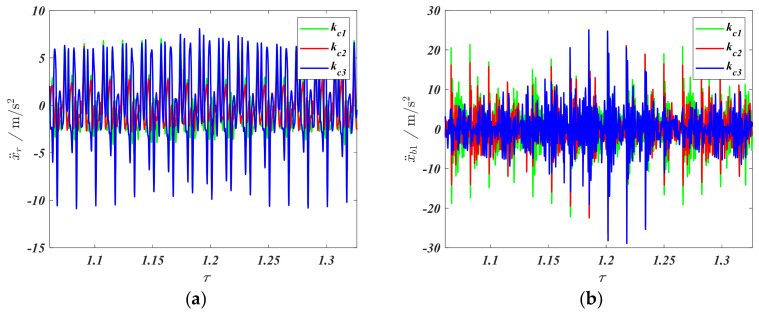
Time-domain vibration acceleration of the system with increasing rub-impact stiffness in the x-direction (where $k_{c1}$:$k_{c}$ = 1 × 10^10^ N/m, $k_{c2}$:$k_{c}$ = 2 × 10^10^ N/m, and $k_{c3}$:$k_{c}$ = 3 × 10^10^ N/m). (**a**) the disk; (**b**) the left bearing.

Figure 21a,b present the x-direction time-domain vibration accelerations of the disk and left bearing, respectively, under increasing rub-impact stiffness. The time-domain analysis shows that higher stiffness subjects the disk to continuous high-amplitude impacts and sustained force interactions. Under severe stiffness constraints, the collision process becomes brief yet more intense, causing the time-domain waveform to evolve from a relatively smooth profile into sharp, periodic impulse-like signals.

**Figure 22:**
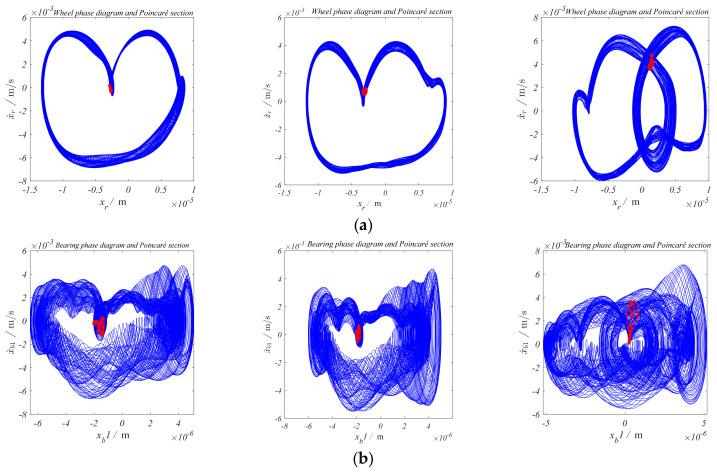
Phase portraits and Poincaré sections of the system with increasing rub-impact stiffness. (**a**) the phase portraits and Poincaré sections of the disk under increasing rub-impact stiffness; (**b**) the phase portraits and Poincaré sections of the left bearing under increasing rub-impact stiffness.

Figure 22a,b present the phase portraits and Poincaré sections of the disk and left bearing, respectively, under increasing rub-impact stiffness. The disk’s phase trajectories preserve the inherent structural features of rub-impact faults. Conversely, the bearing’s phase portraits become increasingly chaotic with rising stiffness, manifested by a wider, more scattered point distribution within the Poincaré sections.

**Figure 23:**
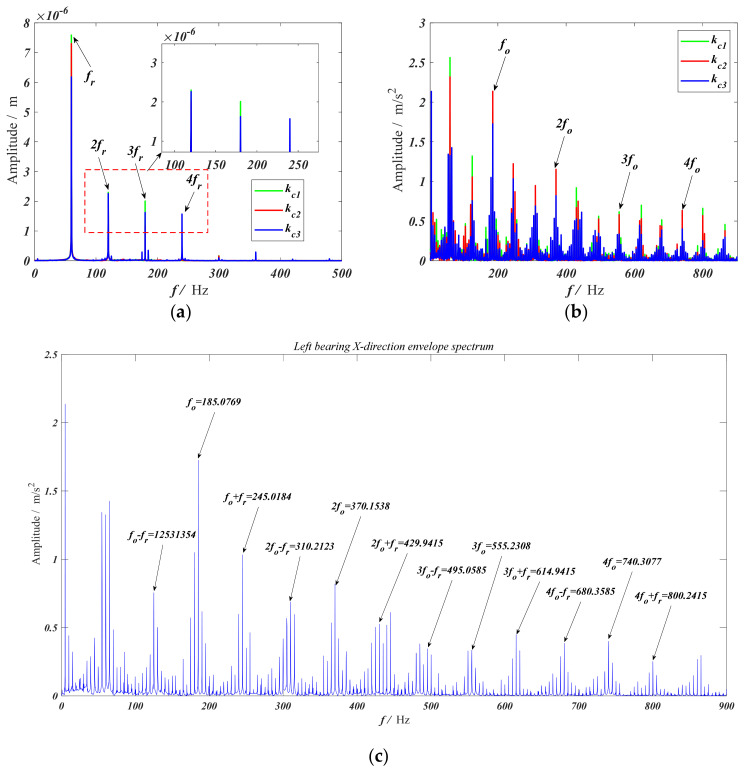
Frequency spectra and envelope spectra of the system with increasing rub-impact stiffness in the x-direction (where $k_{c1}$:$k_{c}$ = 1 × 10^10^ N/m, $k_{c2}$:$k_{c}$ = 2 × 10^10^ N/m, and $k_{c3}$:$k_{c}$ = 3 × 10^10^ N/m). (**a**) the x-direction frequency spectrum of the disk under increasing rub-impact stiffness; (**b**) the envelope spectra of the left bearing under increasing rub-impact stiffness; (**c**) the direct spectra of the left bearing under increasing rub-impact stiffness.

Figure 23a–c present the x-direction frequency spectrum of the disk, alongside the envelope and direct spectra of the left bearing under increasing rub-impact stiffness, where $f_{r}$ and $f_{o}$ denote the fundamental rotational frequency and the outer-race fault characteristic frequency, respectively. At higher stiffness levels, the shortened, more intense collision process induces a prominent fourth harmonic component ($4f_{r}$) in the direct spectrum. Meanwhile, the bearing envelope spectrum consistently captures $f_{o}$ and its modulation components. Overall, the disk dynamics remain heavily dominated by the rub-impact mechanism, whereas the bearing end exhibits increasingly intricate non-linear behavior as the amplified stiffness intensifies rub-induced effects while preserving outer-race fault features.

#### 3.3.2. Dynamic Analysis of the Influence of Fault Width Variation on System Behavior

**Figure 24:**
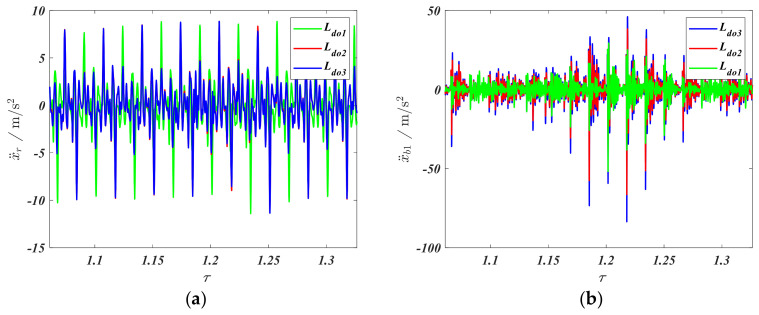
Time-domain vibration acceleration of the system with increasing bearing outer race defect width in the x-direction (where $L_{do1}$:$L_{do}$ = 3 mm, $L_{do2}$:$L_{do}$ = 4 mm, and $L_{do3}$:$L_{do}$ = 5 mm). (**a**) the disk; (**b**) the left bearing.

**Figure 25:**
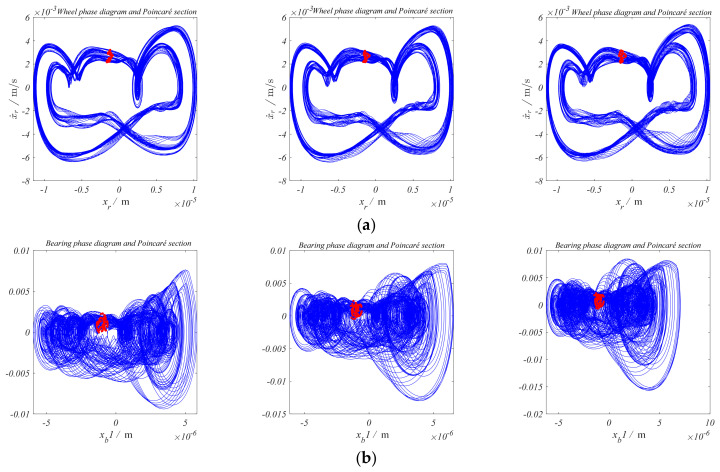
Phase portraits and Poincaré sections of the system with increasing bearing outer-race defect width. (**a**) the time-domain vibration acceleration of the disk end with increasing outer-race defect width; (**b**) the time-domain vibration acceleration of the left bearing end with increasing outer-race defect width.

**Figure 26:**
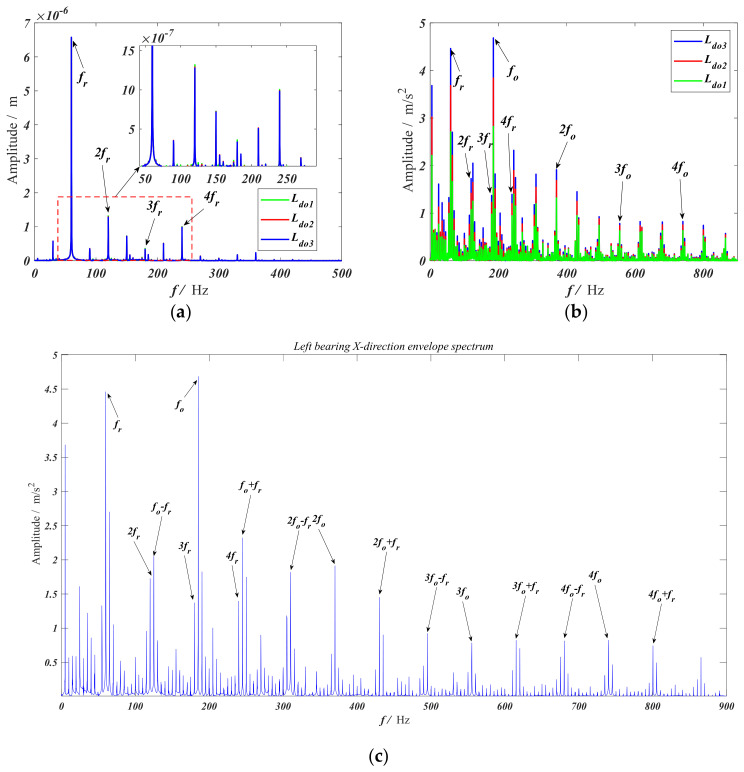
Frequency spectra and envelope spectra of the system with increasing bearing outer-race defect width in the x-direction (where $L_{do1}$:$L_{do}$ = 3 mm, $L_{do2}$:$L_{do}$ = 4 mm, and $L_{do3}$:$L_{do}$ = 5 mm). (**a**) the phase portraits and Poincaré sections of the disk end with increasing outer-race defect width; (**b**) the phase portraits and Poincaré sections of the left bearing end with increasing outer-race defect width; (**c**) direct spectra of the left bearing.

Figure 24, Figure 25 and Figure 26 present the system’s dynamic responses at a constant rotational speed under fixed eccentricity and clearance parameters, with varying bearing outer-race fault widths ($L_{do}$). Comparing these operational states effectively reveals the evolutionary characteristics and underlying mechanisms of the system dynamics induced by outer-race fault expansion.

Figure 25a shows the time-domain vibration acceleration of the disk end with increasing outer-race defect width, while Figure 25b presents that of the left bearing under the same conditions.

Figure 26a shows the phase portraits and Poincaré sections of the disk end with increasing outer-race defect width, while Figure 26b presents those of the left bearing under the same conditions.

Figure 26a–c display the x-direction frequency spectrum of the disk, alongside the envelope and direct spectra of the left bearing under increasing outer-race defect width. The spectral analysis reveals that while the disk-end response exhibits minimal variations, the bearing end shows pronounced sensitivity; the amplitudes of the outer-race fault characteristic frequency ($f_{o}$) and its harmonics increase sharply, emerging as dominant components in the spectrum.

The analysis encompasses the system’s time-domain accelerations, phase trajectories, Poincaré sections, direct spectra, and envelope spectra. Consistent with preceding findings, the outer-race fault exerts a negligible macro-influence on the disk’s non-linear response, which remains predominantly governed by the rub-impact mechanism as the defect width expands. Conversely, the bearing-end vibration amplitude escalates significantly with defect width, accompanied by a sharp amplitude increase in the outer-race fault characteristic frequency and its harmonics within the envelope spectrum. Synthesizing these results, it is concluded that under coupled rubbing–outer-race faults, the disk dynamics primarily reflect the global rubbing state, whereas the bearing response is highly sensitive to local outer-race defects, whose impact features are further intensified by rub-induced modulation. Since the impact energy generated by the outer-race fault gradually attenuates during spatial transmission, the weak fault signatures are easily masked by strong rubbing vibrations, making the outer-race fault frequency components difficult to identify clearly in the disk spectrum. This characteristic frequency masking mechanism and the non-linear spectrum modulation observed under the coupled fault state exhibit strong qualitative alignment with the multi-fault spectral coupling and interactive bifurcation paradigms previously identified by Ma et al. [19]. Meanwhile, the high-frequency localized impulsive spectral responses captured at the bearing end closely adhere to the lumped-discretization fault simulation characteristics validated by Wang et al. [5]. Consequently, these organic cross-validations with the established rotor dynamics literature firmly verify the scientific accuracy, mechanical reliability, and practical diagnostic tracking value of the presented model.

Table 3 compares the bearing outer-race and inner-race fault characteristic frequencies obtained from numerical simulations and theoretical calculations under the same compound fault condition at the investigated operational velocity. The theoretical benchmarks calculated via standard kinematic formulations yield fundamental frequencies of 184.90 Hz for the outer-race and 294.64 Hz for the inner-race. Crucially, the proposed non-linear differential solver extracts these discrete components at 185.10 Hz and 294.99 Hz, respectively. The resulting relative deviation is strictly bounded within an exceptionally low margin below 0.12% (with the maximum tracking error of 0.114% occurring at the second harmonic of the outer-race fault). These highly precise matchings firmly demonstrate that the numerical solution results of the established multi-fault model are highly accurate. The mathematical algorithm possesses extremely high fidelity in tracking discrete impulse modulations, providing an absolute quantitative foundation for the subsequent interactive bifurcation analysis.

## 4. Conclusions

This study focuses on a rolling bearing–rotor system with faults. Based on the classical Jeffcott rotor theory, three different dynamic models were established, and the dynamic characteristics of the rotor system under three operating conditions were analyzed using numerical solutions. Crucially, the obtained numerical trajectories and spectral modulations fully confirm our initial physical expectations and theoretical assumptions; the global continuous rubbing boundaries systematically govern the high-energy states and sequentially mask the micro-scale localized bearing fault signatures. By comprehensively examining time-domain responses, phase trajectories, Poincaré sections, and envelope spectra, the influence of various fault parameters on system dynamic behavior was systematically identified, thereby elucidating the evolutionary mechanisms of rotor dynamics under different fault conditions. The main conclusions are as follows:

(1) Rub-impact fault rotor system: as the rubbing stiffness and eccentricity increase, the system non-linear behavior is significantly enhanced. The time-domain response gradually evolves from a regular sinusoidal signal to irregular vibration, while the shaft orbit transforms from a stable ellipse to a typical flower-shaped trajectory and eventually tends toward full annular rubbing. In the frequency spectrum, sideband and subharmonic components progressively increase, whereas the fundamental frequency amplitude decreases, indicating intensified rubbing-induced impacts and enhanced energy transfer toward higher-frequency components. Notably, the increase in rubbing stiffness not only strengthens non-linear impact behavior but also exhibits a certain suppressing effect on rotor imbalance vibration.
(2) Under rubbing-bearing compound fault conditions, the system exhibits significant non-linear coupling characteristics. As the rubbing stiffness increases, the disk response remains dominated by rubbing behavior, characterized by enhanced time-domain impacts, more complex phase trajectories, and increased higher-order harmonic components, indicating progressively intensified non-linear collision effects. Meanwhile, the bearing response is simultaneously affected by transmitted rubbing excitation and local defects, resulting in more complex dynamic behavior.
(3) As the defect widths of the inner and outer races increase, the bearing response exhibits stronger impact amplitudes in the time domain, higher dispersion in the Poincaré section, and enhanced fault characteristic frequencies and their harmonic components in the envelope spectrum, demonstrating high sensitivity to local defects. In contrast, local fault-induced excitations gradually attenuate during transmission to the disk and are easily masked by the dominant rubbing response, indicating that the disk response mainly reflects the global rubbing condition, whereas the bearing response is more suitable for local fault identification.

In terms of engineering significance, these quantitative boundaries offer a vital parametric baseline for the structural health monitoring and multi-fault isolation of aero-engine and vehicle traction rotors, demonstrating that sensor placement at the bearing housing is far more optimal for early-stage localized fault tracking than shaft-disk displacement monitoring. However, from the perspective of scientific methodology, the developed model contains specific structural limitations: it assumes ideally rigid bodies for the bearing components and simplifies the boundary contacts by neglecting the complex rheological influences of lubricant films and advanced tribological boundary layers. It should be emphasized that the formulation and numerical investigations presented in this study primarily focus on early-stage localized single-raceway defects, serving as a fundamental benchmark for multi-fault rotor systems. In practical engineering applications, the structural health and resulting vibration patterns may exhibit higher complexity if the system escalates to multiple distributed defects or rolling element faults. Under such scenarios, the successive impact modulations, continuous phase variations, and multi-impulse superpositions will alter the established bifurcation paths and chaos boundaries. Future extensions of this model will accommodate these distributed multi-defect topologies to widen its diagnostic applicability. To bridge the gap between this idealized framework and multi-physical engineering realities, future research will be directed towards the following distinct guidelines for experimental validation and model enhancement:

(i) Designing a dedicated double-bearing single-disk rotor test rig equipped with high-precision eddy-current displacement sensors and housing accelerometers to experimentally validate the simulated structural transmission attenuation and frequency masking thresholds;
(ii) Upgrading the current model into a full spatial multi-body dynamics architecture to incorporate angular movements, axial loads, and the dominant gyroscopic moments;
(iii) Introducing rigorous fluid–structure coupling by accounting for lubricant viscosity, squeeze-film damping, and elastohydrodynamic lubrication (EHL) to explore the interactive friction–wear behaviors under non-continuous contact conditions;
(iv) Upgrading the constant friction model into a velocity–temperature-dependent non-linear formulation where local thermal fields and relative sliding kinematics are coupled to dynamically update the friction coefficient under extreme thermal ablation.

## Figures and Tables

**Table 1:** Rolling bearing parameters.

Ball Pitch Diameter (mm)	Ball Diameter d (mm)	Contact Angle T (°)	Number of Balls *N_b_*
104	23.8	0	8

**Table 2:** Rolling bearing fault characteristic frequency.

Bearing Location	Value
outer ring	$f_{o}=\frac{N_{b}}{2}\left(1-\frac{d}{D}cosT\right)f_{r}=3.0846f_{r}$
inner ring	$f_{i}=\frac{N_{b}}{2}\left(1+\frac{d}{D}cosT\right)f_{r}=4.9154f_{r}$

**Table 3:** Comparison of analytical and numerical fault characteristic frequencies.

Bearing Fault Location	Calculated Value	Fundamental Characteristic Frequency (Hz)	Second Harmonic (Hz)	Third Harmonic (Hz)	Fourth Harmonic (Hz)
outer-race fault	theoretical value	184.90	369.79	554.68	739.58
	numerical value	185.10	370.21	555.31	740.22
inner-race fault	theoretical value	294.64	589.27	883.91	1178.54
	numerical value	294.99	589.80	884.79	1179.60

## Data Availability

The data presented in this study are openly available in Twin-Spool Rotor Misalignment, Looseness, and Rub-Impact Vibration Dataset at 10.17632/b6bfd7v86d.2.

## Nomenclature

Symbol	Description	Value (from Solver)	Unit
$v_{i},v_{o},v_{b}$	Linear velocity of the contact between the rolling element and the inner and outer races of the bearing, orbital linear velocity of the cage centroid	Variable	m/s
$\omega_{i},\omega_{o},\omega_{c},\omega$	Angular velocity of the bearing inner-race, angular velocity of the bearing outer race, angular velocity of the cage, rotational speed of the shaft	Variable	Rad/s
$D_{b},D$	Rolling element diameter, outer diameter of the bearing outer race	23.8; 140.0	mm
$d_{i},d_{o},d_{m}$	Respectively denote the raceway diameters of the inner and outer races, and the pitch circle diameter	80.2; 127.8; 104.0	mm
$\varphi_{i}$	Rotational angle of the i-th rolling element within time t	Variable	rad
$N_{b}$	Number of rolling elements	8	
$\theta_{i}$	Angular position of the i-th rolling element	Variable	rad
$f_{\nu c}$	Ball pass vibration frequency	Variable	Hz
Γ,Σ	Depends on the Hertzian contact coefficient characterizing the contact surface properties	Calculated internally	N/m^1.5^
E	Young’s modulus	2.07 × 10^5^	MPa
υ	Poisson’s ratio	0.3	
∑ρ	Sum of the curvature of the contact elements	Calculated internally	mm^−1^
$r_{\theta i}$	Elastic deformation generated by contact between the i-th rolling element and the inner and outer raceways at any time	Variable	μm
k	Shaft restoring stiffness	2.5 × 10^7^	N/m
$k_{b}$	Equivalent contact stiffness coefficient of the bearing	Calculated	N/m
$k_{c}$	Equivalent stator rub-impact stiffness coefficient	Variable	N/m
$f_{bx1,}f_{by1,}f_{bx2,}f_{by2}$	Hertzian total contact force component of the left bearing, Hertzian total contact force component of the right bearing	Variable	N
$f_{ixb1}f_{iyb1}f_{xb2}f_{yb2}$	Resultant Hertzian contact force components in the x- and y-directions of the left and right bearings when an inner-race defect exists only in the left bearing	Variable	N
$O_{1},O_{2},O_{3}$	Geometric center of the bearing, geometric center of the rotor, mass center of the rotor		
$m_{r},m_{b1},m_{b2}$	Total mass of the rotor; equivalent mass at the left bearing; equivalent mass at the right bearing; disk mass	32.1; 4; 4	kg
$c_{b},c_{r},c_{b1},c_{b2}$	Damping coefficients at the bearing locations; damping coefficient at the rotor disk; damping coefficient at the left bearing end; damping coefficient at the right bearing end	-; 2100.0; 1050.0; 1050.0	N∙s/m
$P_{T},P_{N},P_{x,}P_{y}$	Tangential friction force induced by rub-impact; normal contact force induced by rub-impact; rub-impact force in the x-direction; rub-impact force in the y-direction	Variable	N
$r,r_{i},r_{o}$	Shaft center displacement; outer diameter of the inner race; inner diameter of the outer race		
δ	Rub-impact clearance	9.5	μm
f	Friction coefficient	0.1/0.2	
e	$Mass\;eccentricity\;of\;the\;rotor\;disk,e=O_{2}O_{3}$	Variable	mm
$f_{o},f_{i},f_{b},f_{R}$	Outer-race fault frequency, inner-race fault frequency, and ball fault frequency of the rolling bearing; rotational frequency of the rotor	Variable	Hz
$L_{\mathrm{di}},L_{\mathrm{do}},h_{\mathrm{di}},h_{\mathrm{do}}$	Inner-race defect diameter, outer-race defect diameter, inner-race defect depth, outer-race defect depth	Variable	mm
$\Delta h_{\mathrm{di}},\Delta h_{\mathrm{do}}$	Actual depth of the pit engaged by the rolling element in the inner-race defect region; actual depth of the pit engaged by the rolling element in the outer-race defect region	Variable	mm
$\theta_{ie},\theta_{id}\theta_{oe},\theta_{od}$	Half of the central angle corresponding to the inner-race defect region; rotational angle representing the center of the defect region; half of the central angle corresponding to the outer-race defect region; rotational angle representing the center of the defect region	Variable	rad
$R_{b}$	Rolling element radius	11.9	mm
τ	Dimensionless time constant	Variable	
$F_{xb1},F_{yb1},F_{xb2},F_{yb2}$	Dimensionless resultant contact force components of the left and right bearings	Variable	N
γ	Bearing radial clearance	Variable	μm
